# Notch interaction with RUNX factors regulates initiation of the T-lineage program

**DOI:** 10.1084/jem.20250911

**Published:** 2025-12-04

**Authors:** Yuichi Kama, Ken-ichi Hirano, Kaori Masuhara, Yusuke Endo, Yuka Suzuki, Masanori Fujimoto, Tatsuma Matsuda, Takashi Yahata, Masahiko Kato, Katsuto Hozumi, Tomoaki Tanaka, Hiroyuki Hosokawa

**Affiliations:** 1 Department of Immunology, Tokai University School of Medicine, Isehara, Japan; 2 Department of Pharmacology and Regenerative Medicine, University of Illinois at Chicago, Chicago, IL, USA; 3 Laboratory of Medical Omics Research, Kazusa DNA Research Institute, Kisarazu, Japan; 4 Department of Omics Medicine, Graduate School of Medicine, Chiba University, Chuo-ku, Japan; 5 Department of Molecular Diagnosis, Graduate School of Medicine, Chiba University, Chuo-ku, Japan; 6 Translational Molecular Therapeutics Laboratory, Division of Host Defense Mechanism, Tokai University School of Medicine, Isehara, Japan; 7 Institute of Medical Sciences, Tokai University, Isehara, Japan; 8 Tokai University School of Medicine, Isehara, Japan

## Abstract

Runt-related transcription (RUNX) factors play a key role in T cell development. At the T-lineage commitment checkpoint, RUNX1 undergoes dynamic partner switching, resulting in its redeployment. Here, we investigated the functional differences in RUNX factors between the lymphoid progenitor (LP)– and Notch-stimulated earliest T progenitor stages (Phase 1). We identified CCCTC-binding factor (CTCF) as an LP-specific RUNX1-interacting partner, with LP-specific RUNX1-binding genomic sites significantly enriched for CTCF consensus motifs and co-occupied by CTCF. On Notch stimulation, Notch1 intracellular domain directly interacts with RUNX1 and recruits the RUNX1/Mediator/p300 transcriptional activation complex to Notch-regulated T-signature gene loci. CRISPR/Cas9-mediated stage-specific deletion of RUNX factors and their binding partners revealed that the RUNX1/CTCF complex in LP negatively regulates T-signature gene expression, whereas the RUNX1/Mediator/p300 complex in Phase 1 promotes it. Our findings highlight the crucial role of Notch-mediated functional conversion of RUNX factors, including protein complex reorganization and genomic redeployment in initiating T-lineage program.

## Teaser

The functional dynamics of the Runt-related transcription (RUNX) factors in initiating T-lineage programs remain unclear. Kama et al. show that Notch-mediated reorganization of RUNX complexes and redeployment of RUNX-binding genomic regions are crucial for triggering the T-lineage program.

## Introduction

Runt-related transcription factors (TFs) (RUNX1, RUNX2, and RUNX3) play fundamental roles in various developmental processes by activating or repressing their target genes. These bifunctional TFs require an interacting partner, the core-binding factor subunit beta (Cbfβ), for DNA binding and for the recruitment of coactivators such as p300 or corepressors, e.g., transducin-like enhancer of split/Groucho (de Bruijn and Dzierzak, 2017; Hoogenkamp et al., 2009; Levanon and Groner, 2004; Mevel et al., 2019; Seo and Taniuchi, 2020). Members of the RUNX family often exhibit reciprocal tissue expression patterns, thus limiting functional redundancy (Fukushima-Nakase et al., 2005; Goyama et al., 2004; Levanon et al., 2001). RUNX1 and RUNX3 are known to play important roles in hematopoiesis and T cell development (Collins et al., 2009; Shin et al., 2021; Wang et al., 1996). Notably, RUNX consensus motifs are consistently enriched near genomic regions occupied by key lineage-specifying TFs in multiple hematopoietic lineages (Hosokawa et al., 2021a; Hosokawa et al., 2020; Hosokawa et al., 2018a; Hosokawa et al., 2018b; Miyazaki et al., 2017; Pham et al., 2013; Ungerbäck et al., 2018; Van de Walle et al., 2016).

A small subset of lymphoid-primed multipotent progenitors (LMPPs) migrate from the bone marrow (BM) to the thymus, where Notch signaling triggers their T-lineage program. LMPPs express Notch receptors, whereas thymic epithelial cells provide the Notch ligand, Delta-like 4 (DLL4) (Hozumi et al., 2008; Radtke et al., 1999; Romero-Wolf et al., 2020). The earliest T cell progenitors in the thymus lack the mature T cell markers, CD4 and CD8, classifying them as “double-negative (DN)” thymocytes. DN thymocytes are further subdivided into several phenotypically distinct developmental stages (DN1 to DN4) based on the expression of surface markers, Kit, CD44, and CD25 (Hosokawa and Rothenberg, 2021; Yui and Rothenberg, 2014). Although Notch signaling drives the T-lineage program, early DN1 and DN2a thymocytes retain the potential to differentiate into non–T cell lineages, including innate lymphoid and myeloid cells, reflecting an uncommitted multipotent state. During the transition from DN2a to DN2b, T progenitors lose multipotency and become intrinsically committed to the T lineage, though Notch signaling remains essential for their progression to the next stage (DN3). DN2b and DN3 are thus Notch-dependent, T lineage–committed stages preparing for *Tcrb* (T cell receptor beta chain) gene rearrangement. Thus, early pro-T cells in the thymus, which lack pre–T cell receptor (TCR) expression, can be divided into two Notch-dependent phases: (1) “Phase 1,” precommitment stage (DN1 and DN2a), and (2) “Phase 2,” a post-T lineage–committed stage (DN2b and DN3) (Hosokawa et al., 2021b; Hosokawa and Rothenberg, 2018; Hosokawa and Rothenberg, 2021; Yui and Rothenberg, 2014).

RUNX1 is the predominant RUNX family member involved in early T cell development. In contrast, lymphoid progenitor (LPs) express *Runx2* and *Runx3* mRNA, but their levels decrease or disappear after Notch signaling activation (Shin et al., 2021; Yoshida et al., 2019). Although RUNX1 maintains relatively stable expression kinetics through all T-developmental stages, its function changes dynamically across the T-lineage commitment checkpoint, from the precommitment phase (Phase 1) to the T lineage–committed phase (Phase 2) (Hosokawa et al., 2021b; Hosokawa and Rothenberg, 2021; Shin et al., 2021). In Phase 1, the TF purine-rich box1 (PU.1) predominantly regulates RUNX1 DNA-binding site choices via the recruitment of RUNX1 to target loci. This outcome prevents RUNX1 from occupying T-lineage signature loci and inhibits premature activation of the T-identity program (Hosokawa et al., 2018b; Ungerbäck et al., 2018). In Phase 2, the T-lineage determination TF B cell lymphoma/leukemia 11B (Bcl11b) not only activates T-lineage signature genes through its coactivator RUNX1 but also restricts access to alternative cell fates by sequestering RUNX1 away from lineage-specific loci associated with other developmental pathways (Hosokawa et al., 2018a). Therefore, stage-specific redeployment of RUNX1 by key partner TFs plays a crucial role in the T-lineage commitment checkpoint (Hosokawa et al., 2021b; Hosokawa and Rothenberg, 2021). However, functional differences of RUNX factors in pre-Notch LPs vs. post-Notch Phase 1 cells and the mechanisms underlying context-specific RUNX functions in this developmental transition remain unclear; this is due to the rarity and heterogeneity of LPs *in vivo*.

Here, we evaluated the functional dynamics of RUNX factors before and after the initiation of the T-lineage program as driven by Notch stimulation. We first established early B cell factor 1 (*Ebf1*)–deficient LP cell lines from Cas9 knock-in mice using previously described methods (Hirano et al., 2021; Koizumi et al., 2022; Medina et al., 2004; Pongubala et al., 2008; Zandi et al., 2008). These Cas9-expressing LP (Cas9-LP) cell lines retain the potential to differentiate into mature T cells both *in vitro* and *in vivo*, and exhibit physiological gene expression profiles at the single-cell level. Using Cas9-LPs, we observed dynamic switching of RUNX1-binding sites across the genome in response to Notch stimulation. Notably, the motif for the TF CCCTC-binding factor (CTCF) was highly enriched in LP-specific RUNX1-binding sites, despite being absent in Phase 1–specific RUNX1-binding regions. Proteomic analysis of RUNX1-interacting molecules in LPs and Phase 1 cells identified CTCF as an LP-specific binding partner for RUNX1. Stage-specific deletion of *Cbfb* or RUNX1-interacting molecules revealed that the RUNX1/CTCF complex suppresses the spontaneous activation of T-lineage signature genes in LPs. Moreover, Notch-mediated dissociation of RUNX1 from the CTCF complex, along with redeployment of the RUNX1/Mediator/p300 complex, is involved in the Notch-mediated activation of T-lineage signature genes. Therefore, Notch-mediated functional conversion of RUNX TFs plays a significant role in the initiation of the T-lineage program.

## Results

### Physiological gene expression profiles of Cas9-expressing *Ebf1*-deficient LP cell lines

**Figure S1.**
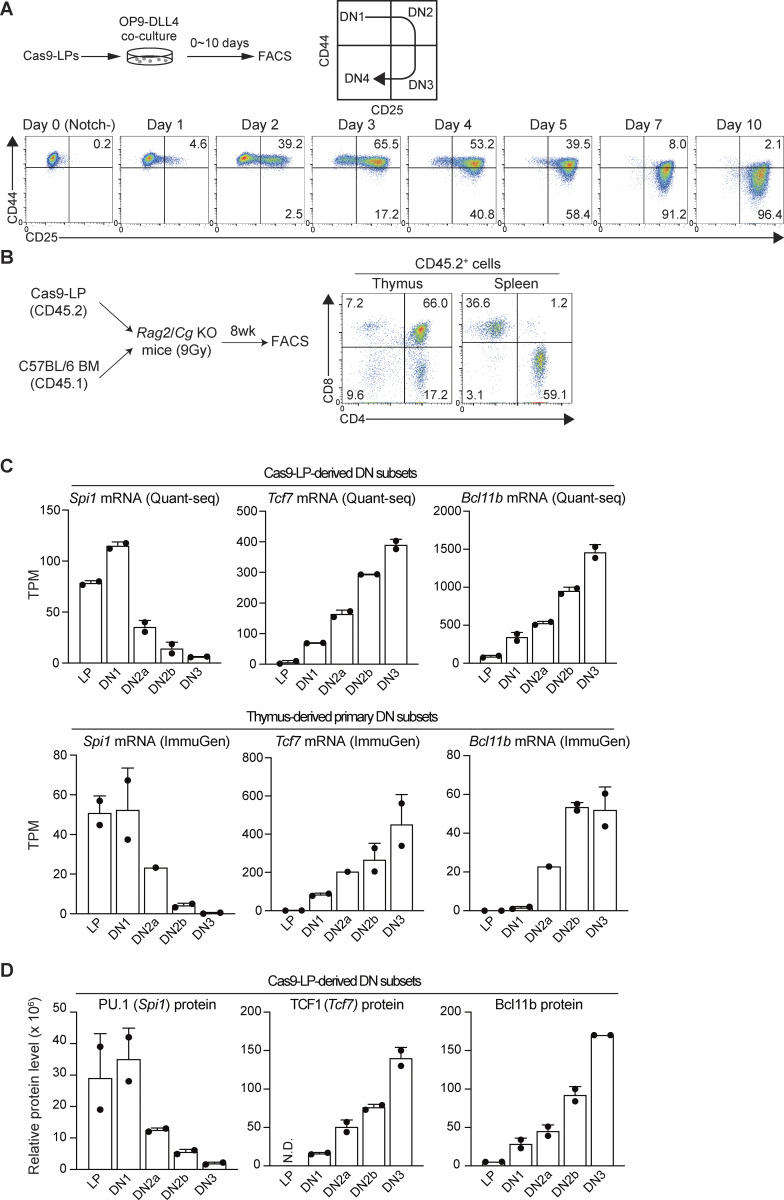
**T cell developmental analysis in Cas9-LPs. (A)** Experimental scheme (upper panel). Cas9-LPs were cocultured with OP9-DLL4, and the T cell developmental status was scored using the T-progenitor markers CD44 and CD25 among CD45^+^ cells at different time points after Notch stimulation (lower panel). **(B)** Cas9-LPs (5 × 10^6^, CD45.2) were mixed with BM cells from wild-type C57BL/6 congenic mice (2 × 10^5^, CD45.1) and transplanted into lethally irradiated (9 Gy) *Rag2*- and *Cg*-deficient lymphopenic hosts. Recipient mice were analyzed 8 wk after transplantation. Flow cytometric analysis of the thymocytes and splenocytes was performed. The representative CD4 and CD8 profiles of CD45.2^+^ thymocytes (left) and splenocytes (right) are shown. Results are representative of three independent biological replicates. **(C)** LP and DN subsets derived from Cas9-LPs were subjected to transcriptome analysis. TPM values for *Spi1*, *Tcf7*, and *Bcl11b* in DN subsets from Cas9-LP (upper) and *in vivo* thymic DNs (lower) are shown with SD (https://www.immgen.org; GSE100738) (Yoshida et al., 2019). **(D)** LP and DN subsets derived from Cas9-LPs were subjected to untargeted proteome analysis. Relative protein levels for PU.1, TCF1, and Bcl11b are shown, including their SDs. SD, standard deviation.

**Figure 1.**
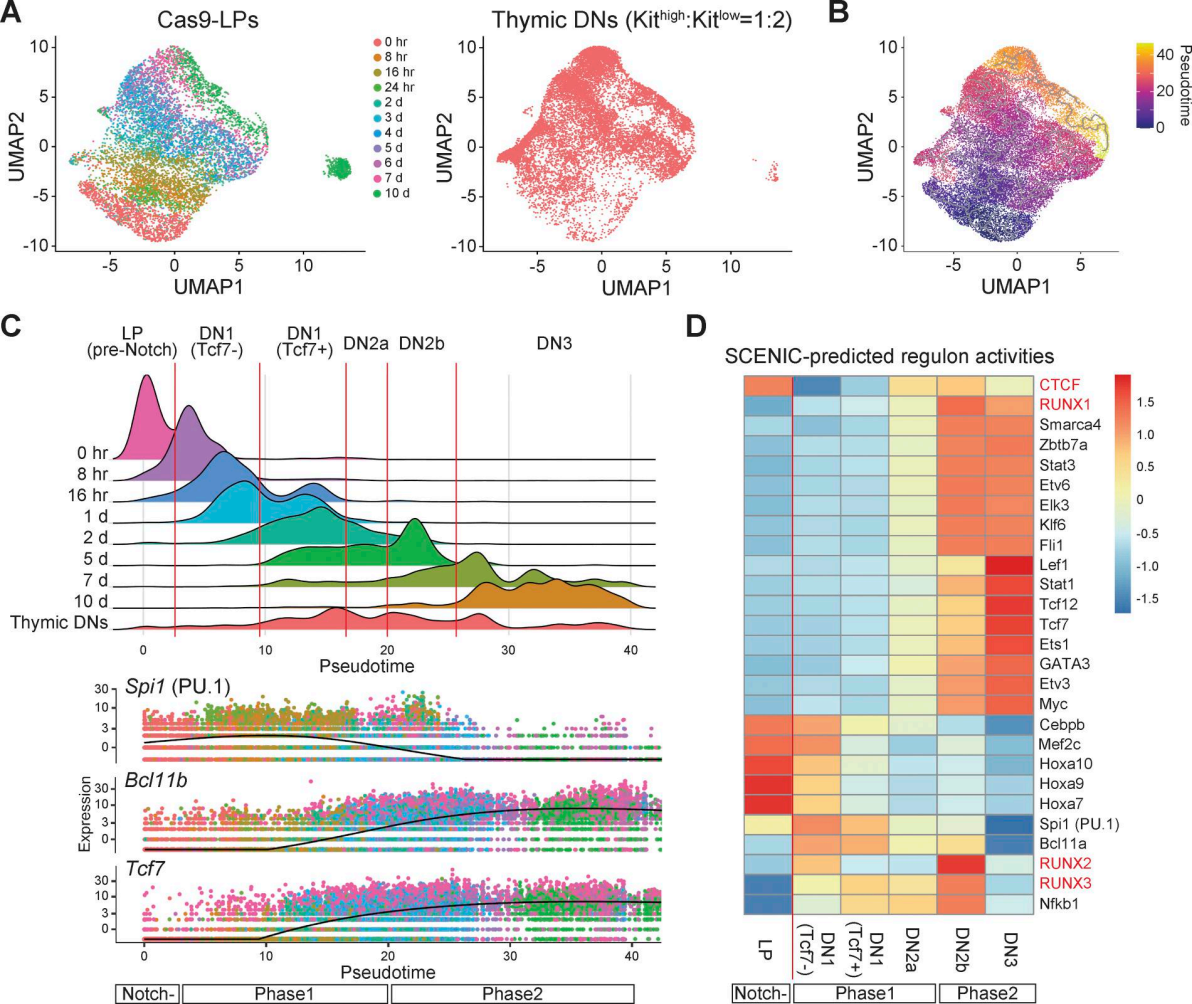
**scRNA-seq reveals transcriptional dynamics of Notch-stimulated LPs. (A)** UMAP visualization of scRNA-seq data from Cas9-LPs with or without Notch stimulation (left) and thymus-derived primary DN cells (Kit^high^: Kit^low^ = 1:2) (right). Clustering was performed using a nonlinear representation of the top 50 principal components. Cells are colored according to the time after Notch stimulation (left). **(B)** Pseudo-time scores of individual cells are shown in UMAP1-2, with colors indicating progression along pseudo-time. Pseudo-time scores were calculated using Monocle3, with the principal root node defined as LPs without Notch stimulation. **(C)** Distribution of Cas9-LPs, Notch-stimulated T progenitors, and primary thymic DN cells along pseudo-time (top). The relative expression patterns of *Spi1*, *Bcl11b*, and *Tcf7* across pseudo-time are shown (bottom). **(D)** Heatmap showing SCENIC-predicted regulon activities of the indicated TFs across different developmental stages of DN cells. Data are based on two independent experiments, and pooled data were used for analysis.

**Figure S2.**
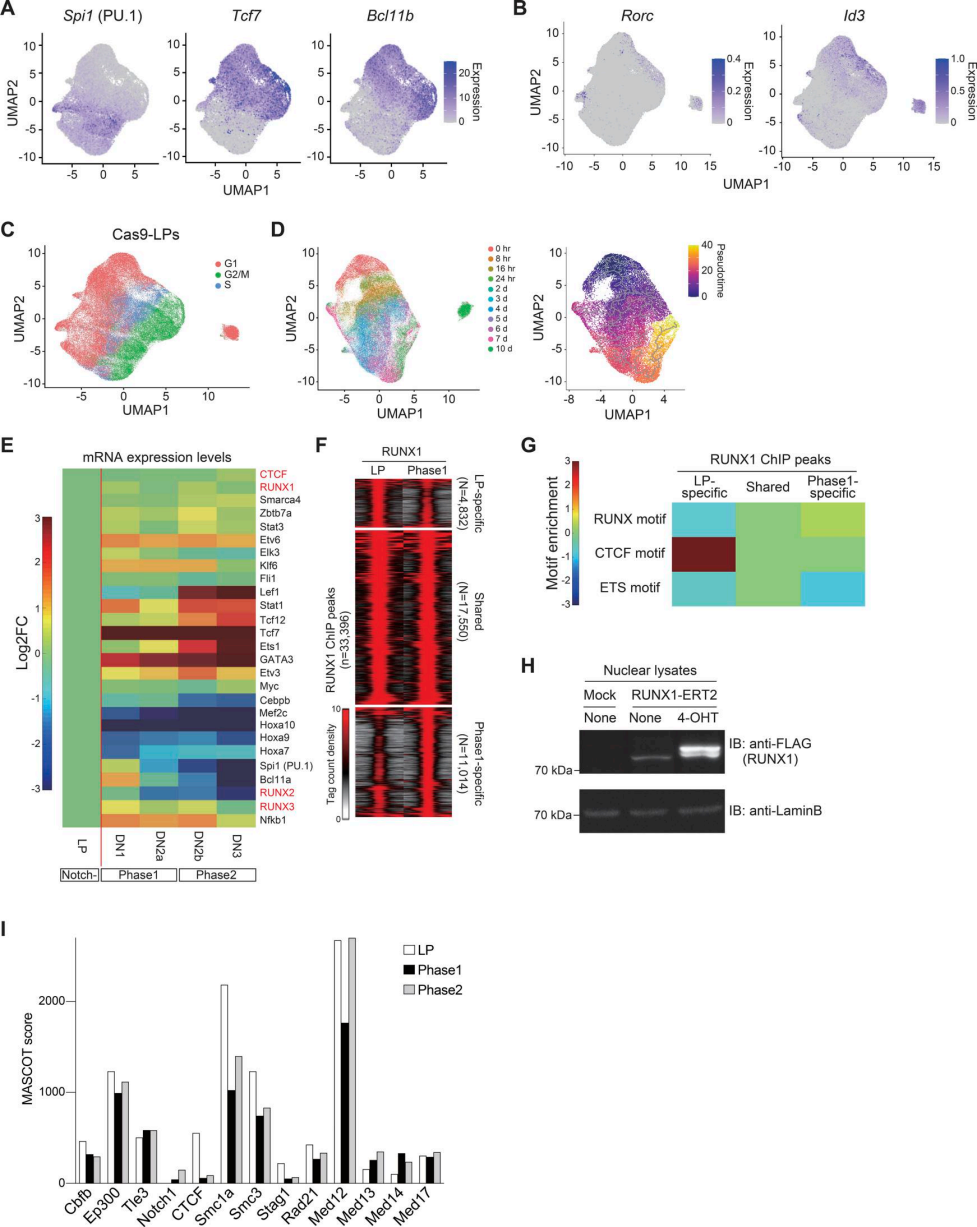
**Validation of T cell development in Cas9-LPs at the single-cell level. (A)** UMAP1-2 visualization of scRNA-seq data from Cas9-LP with or without Notch stimulation (Fig. 1 A, left). The color intensity represents the expression levels of *Spi1*, *Tcf7*, and *Bcl11b*, which indicate the progression of T cell development. **(B)** UMAP1-2 visualization of scRNA-seq data from Cas9-LP with or without Notch stimulation (Fig. 1 A, left). The color intensity indicates the expression levels of *Rorc* and *Id3*, which are informative for identifying Phase 3 cells. **(C)** UMAP visualization of scRNA-seq data for Cas9-LP with or without Notch stimulation (related to Fig. 1 A, left). The cells are colored according to the cell cycle stages. **(D)** UMAP visualization of scRNA-seq data for Cas9-LPs with and without Notch stimulation. Clustering was performed using a nonlinear representation of the top 50 principal components, excluding PC2, which contained many cell cycle–related genes. The cells are colored according to the time after Notch stimulation (left) or the pseudo-time scores (right). **(E)** Heatmap showing changes in the expression of TFs in Fig. 1 D across different developmental stages of DN cells. **(F)** Tag count distributions for RUNX1 ChIP signals are shown as peak-centered heatmaps. Each lane represents the merged tag directories from two biological replicates. **(G)** Heatmap showing changes in the motif enrichment of stage-specific RUNX1-binding genomic regions in Fig. 2, A and B. **(H)** Myc- and FLAG-tagged RUNX1-ERT2 were retrovirally transduced into Cas9-LPs, and cells were treated with tamoxifen for 6 h. Nuclear lysates from Mock- or Myc-FLAG-RUNX1-ERT2–expressing LPs, with or without tamoxifen treatment, were subjected to immunoblotting with anti-FLAG and anti-lamin B antibodies. Two independent experiments were performed with similar results. **(I)** Mascot scores of representative RUNX1-binding molecules in LP, Phase 1, and Phase 2 cells (Fig. 2 D) are shown. Source data are available for this figure: SourceData FS2.

*Ebf1*-deficient LP cell lines, established by multiple research groups (Hirano et al., 2021; Koizumi et al., 2022; Medina et al., 2004; Pongubala et al., 2008; Zandi et al., 2008), are useful tools for characterizing the roles of TFs in LPs and early T cell progenitors (Hirano et al., 2021; Koizumi et al., 2022). During our attempt to delete the target genes using the CRISPR/Cas9 system, we found that retrovirus-mediated overexpression of Cas9 was highly toxic to *Ebf1*-deficient LP cell lines (Koizumi et al., 2022). To overcome this limitation, we generated Cas9-expressing *Ebf1*–deficient LP (Cas9-LP) cell lines from Rosa26-Cas9 knock-in mice, which express low but sufficient levels of the Cas9 protein. Cas9-LP lines retained the potential to differentiate into T cells both *in vitro* and *in vivo*, as indicated by the expression of key cell surface markers (Fig. S1, A and B). Transcriptome and untargeted proteome analyses (Kanno et al., 2023), using DN subsets derived from Notch-stimulated Cas9-LPs, showed that mRNA and protein expression kinetics of key TFs, including Spi-1 proto-oncogene (*Spi1*, encoding PU.1), Tcf7 (encoding T cell factor-1, TCF1), and Bcl11b, were comparable with those of *in vivo* thymic DN subsets (Fig. S1, C and D) (Yoshida et al., 2019). To determine whether Cas9-LPs exhibit physiological gene expression profiles at the single-cell level, we performed single-cell RNA sequencing (scRNA-seq) at different time points following the *in vitro* stimulation of Notch (0, 8, 16, and 24 h, and 2, 3, 4, 5, 6, 7, and 10 days). Additionally, we analyzed primary DN subsets isolated from the thymus. Considering the small number of primary Phase 1 DN thymocytes—DN1 (CD44^+^Kit^high^ CD25^−^) and DN2a (CD44^+^Kit^high^ CD25^+^)—we sorted and pooled CD44^+^Kit^high^ Phase 1 and CD44^+^Kit^low^CD25^+^ Phase 2 cells at a 1:2 ratio for further analysis. Data from two independent experiments were pooled and analyzed. Specifically, after dimension reduction and clustering, Notch-stimulated Cas9-LPs and *in vivo* thymic DNs clustered together, exhibiting remarkably similar gene expression kinetics for critical landmark genes, namely, *Spi1* (highly expressed in Phase 1), *Tcf7* (induced in Phase 1 and increased in Phase 2), and *Bcl11b* (induced at the T-lineage commitment) (Fig. 1 A and Fig S2 A). Although enrichment of Phase 1 cells from DN thymocytes provided limited resolution of the earliest Notch-stimulated Phase 1 cells, early time points in Notch-stimulated Cas9-LPs clearly showed a gradual progression of T cell development, as indicated via pseudo-time analysis (Fig. 1, A and B) (Hosokawa and Rothenberg, 2021; Shin et al., 2024; Yui and Rothenberg, 2014; Zhou et al., 2022; Zhou et al., 2019). A small cluster with the highest Uniform Manifold Approximation and Projection (UMAP1) values consisted primarily of Cas9-LPs stimulated with Notch for 10 days, and expressing RAR-related orphan receptor C (*Rorc*) and inhibitor of DNA binding 3 (*Id3*) (Fig. 1 A and Fig. S2 B). This cluster likely represents Phase 3 cells, which have passed the β-selection checkpoint (Taghon et al., 2006; Xi et al., 2006; Yashiro-Ohtani et al., 2009). Cell cycle projections showed that UMAP1 values correlated with cell cycle phases (Fig. S2 C). To ensure that pseudo-time progression reflected cellular state transitions rather than cell cycle effects, we regressed cell cycle scores. Even after adjusting for cell cycle effects, the clear progression of T cell development was evident (Fig. S2 D). Collectively, these results demonstrate that Notch-stimulated Cas9-LP cell lines closely recapitulate early thymic T progenitors *in vivo* at the single-cell transcriptome level, thereby establishing a useful tool for dissecting molecular events underlying the initiation of the T-lineage program.

### High CTCF regulon activity in LPs diminishes shortly after Notch stimulation

As the Cas9-LP system provides a new opportunity to track the first steps of the T-lineage program at a fine resolution, we worked to elucidate the TFs that underlie this process. Based on the expression of key TFs and pseudo-time progression, cells were categorized as DN1, DN2a, DN2b, or DN3 (Fig. 1 C). Notably, pseudo-time progression began within 8 h of Notch stimulation, preceding the activation of one of the earliest Notch target genes, *Tcf7* (Weber et al., 2011). To account for this, DN1 cells were further divided into two substages based on the presence or absence of *Tcf7* expression (Fig. 1 C). Using these DN subset classifications, we applied single-cell regulatory network inference and clustering (SCENIC) to identify TFs with dynamic activity based on the expression patterns of their putative target genes. The expression of a TF does not always correlate with its activity. SCENIC is a computational framework used to infer actual TF activity based on downstream target gene expression. The activity of progenitor-associated regulons, including *Spi1* (PU.1), myocyte enhancer factor 2C (*Mef2c*), and homeobox A9 (*Hoxa9*), gradually declined following Notch stimulation, whereas T progenitor–associated regulons, such as *Tcf7*, GATA-binding protein 3 (*Gata3*), *Tcf12*, and lymphoid enhancer-binding factor 1 (*Lef1*) regulons, progressively increased, consistent with their expression kinetics (Fig. 1 D and Fig. S2 E). The activity of RUNX family regulons exhibited a modest increase at the DN1 (Tcf7^−^) stage following Notch stimulation. Notably, the CTCF regulon, which exhibited high activity in LP, showed a dramatic decrease at the DN1 (Tcf7^−^) stage (Fig. 1 D). Importantly, expression levels of RUNX family members and CTCF were comparable before and after Notch stimulation (Fig. S2 E) (Shin et al., 2021; Yoshida et al., 2019), indicating that the observed changes in regulon activity were likely attributable to functional modulation rather than alterations in gene expression levels. Therefore, gene regulatory network inference suggests that Notch-mediated initiation of T-lineage program results in the attenuation of CTCF and the elevation of RUNX activity in LPs.

### RUNX1 shifts its genomic binding sites between LP and Phase 1 cells

**Figure 2.**
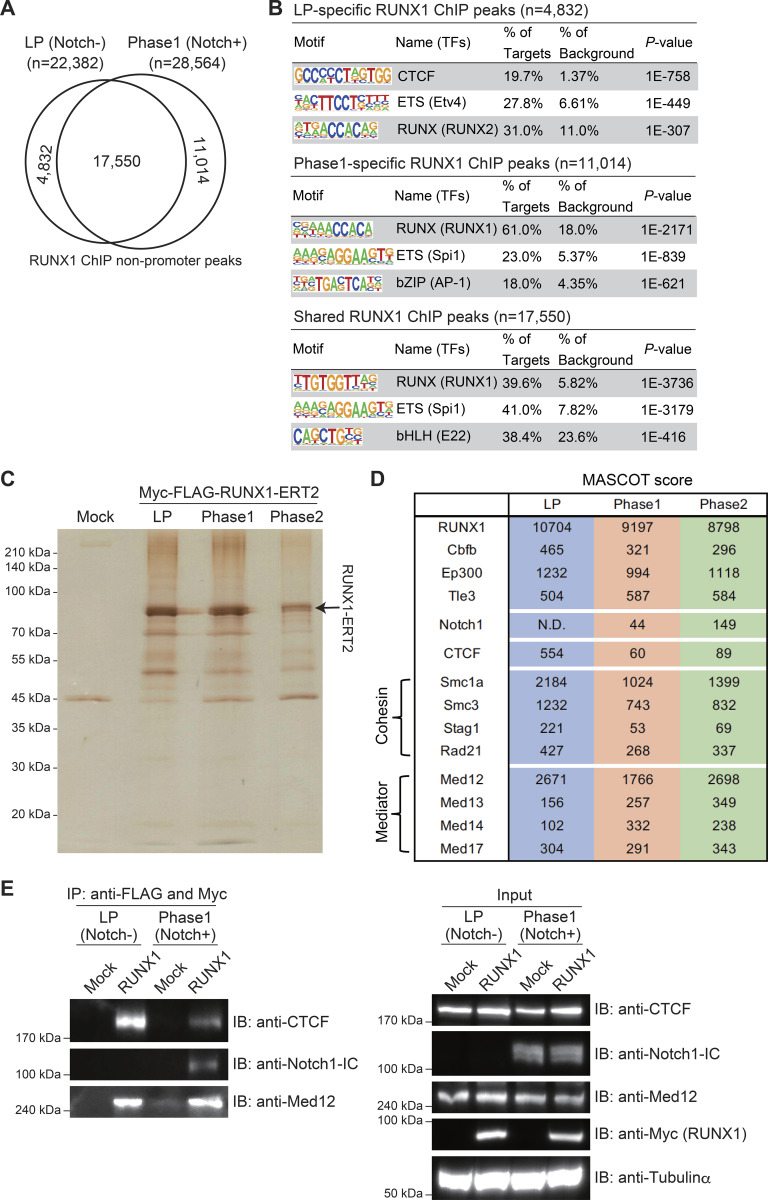
**CTCF motif is highly enriched in LP-specific RUNX1-binding regions and LP-specific interaction of RUNX1 with CTCF. (A)** ChIP-seq analysis of RUNX1 was performed using Cas9-LPs with or without Notch stimulation. The Venn diagram shows the numbers of reproducible RUNX1 nonpromoter ChIP peaks in LPs (Notch-) and Notch-stimulated LPs for 2 days (Phase 1; Notch+). **(B)** Top three enriched sequence motifs among the 4,832 LP-specific, the 11,014 Phase 1–specific, and 17,550 shared reproducible RUNX1 peaks between LP and Phase 1 are shown. Data are based on ChIP-seq peaks scored as reproducible in two replicate samples. **(C)** Myc- and FLAG-tagged RUNX1-ERT2 vectors were retrovirally transduced into Cas9-LPs. Total extracts from Myc-FLAG-RUNX1-ERT2–expressing LPs treated with tamoxifen for 6 h were subjected to two-step affinity purification followed by SDS-PAGE and silver staining. All of the visible bands were analyzed using mass spectrometry analysis. Phase 1 and Phase 2 cells were stimulated with Notch ligand on OP9-DLL4 for 2 and 10 days, respectively. **(D)** Representative RUNX1-binding molecules in LP, Phase 1, and Phase 2 cells are shown with Mascot scores. The full list of RUNX1-binding molecules is provided in Table S1. **(E)** Total extracts from Mock- or Myc-FLAG-RUNX1-ERT2–transduced and tamoxifen-treated LPs, with or without Notch stimulation, were subjected to IP with anti-FLAG and anti-Myc mAbs followed by immunoblotting with anti-CTCF, anti-Notch1-IC, or anti-Med12 antibodies (left panels). Nuclear lysates (input) were also immunoblotted with anti-CTCF, anti-Notch1-IC, anti-Med12, and anti-Myc (RUNX1) antibodies, whereas cytoplasmic lysates (input) were immunoblotted with anti-tubulin-α mAb (right panels). Data are representative of three independent experiments. IP, immunoprecipitation. Source data are available for this figure: SourceData F2.

**Figure S3.**
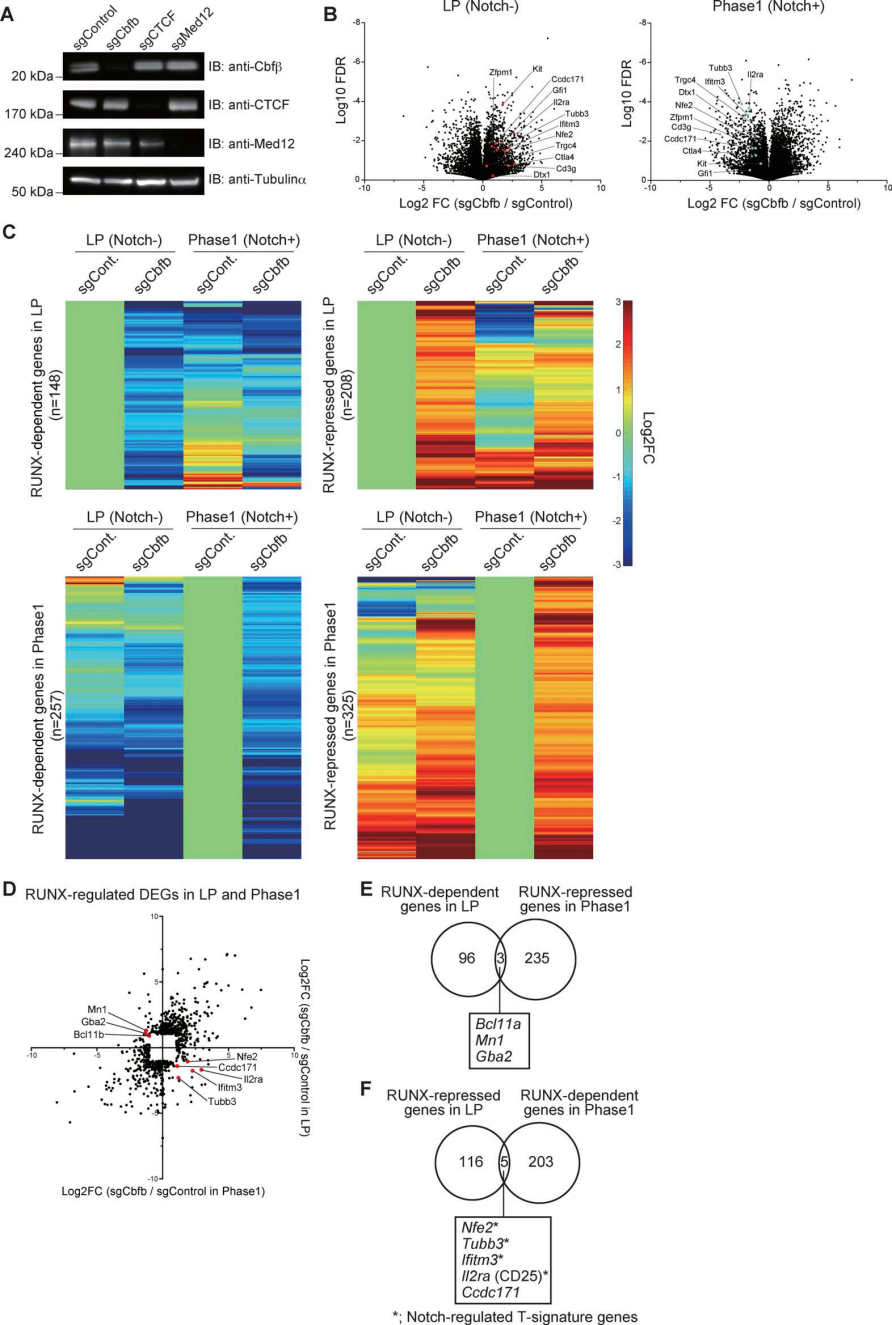
**Validation of RUNX1, CTCF, and Med12 depletion and identification of RUNX1-regulated genes in LP and Phase 1 cells. (A)** sgRNAs against *Cbfb*, *Ctcf*, or *Med12* were introduced into Cas9-LPs. 3 days after sgRNA transduction, nuclear lysates from retrovirus-infected hNGFR^+^ cells were subjected to immunoblotting for Cbfβ, CTCF, and Med12 antibodies, while cytoplasmic lysates were subjected to immunoblotting with anti-tubulin-α mAb. **(B)** Volcano plots showing changes of transcriptome profiles between control and *Cbfb*-deficient LP (left) and Phase 1 cells (right). **(C)** Heatmap showing changes in the expression of RUNX-dependent and RUNX-repressed genes in LP and Phase 1 following *Cbfb* deletion. **(D)** Dot plot showing expression changes of RUNX-regulated DEGs (Fig. 3 B) in LP and Phase 1 cells following the disruption of *Cbfb*. **(E)** Venn diagrams showing the number of RUNX-dependent genes in LP and RUNX-repressed genes in Phase 1 (Fig. 3 B). Names of the three overlapping genes are shown. **(F)** Venn diagrams showing the number of RUNX-repressed genes in LP and RUNX-dependent genes in Phase 1 (Fig. 3 B, yellow areas). Names of the five overlapping genes are shown. Two independent experiments were performed with similar results (A). Data are presented as the average of three biological replicates (B–F). Source data are available for this figure: SourceData FS3.

We previously reported that RUNX TFs undergo dynamic shifts in their genomic binding sites and target genes during T-lineage commitment (Shin et al., 2021). However, the functional transition of RUNX1 between LPs and Notch-stimulated Phase 1 cells remains unclear. To investigate this, we utilized highly tractable Cas9-LP cell lines, which exhibit physiological gene expression profiles both before and after Notch stimulation (Fig. 1). We first performed chromatin immunoprecipitation, followed by deep sequencing (ChIP-seq) to analyze RUNX1 binding in LPs and Phase 1 cells (LPs stimulated with Notch for 2 days). As previous studies have reported that RUNX1 occupancy near promoters is weakly associated with their target genes (Shin et al., 2021), we excluded promoter-associated binding sites from our analysis. Among the 22,382 and 28,564 reproducible nonpromoter RUNX1-binding peaks identified in LPs and Phase 1 cells, respectively, 4,832 LP-specific and 11,014 Phase 1–specific RUNX1 peaks were detected across the genome (Fig. 2 A and Fig. S3 F). Notably, motif analysis of stage-specific RUNX1-binding peaks revealed that the CTCF motif was the most highly enriched motif in LP-specific RUNX1-binding sites, whereas the RUNX motif was the third most enriched (Fig. 2 B, top, and Fig. S2 G). In contrast, Phase 1–specific and shared RUNX1-binding sites were highly enriched for RUNX and PU.1 (encoded by the *Spi1*) motifs, with no significant enrichment of the CTCF motif (Fig. 2 B, middle and bottom, and Fig. S2 G). Thus, RUNX1 initially binds to CTCF-associated sites in LPs but subsequently redirects its binding to RUNX and PU.1 motifs on Notch stimulation, enabling transition to initiate the T-lineage program.

### Complex switching from RUNX1/CTCF to RUNX1/Notch1-IC via Notch stimulation

To elucidate the molecular mechanisms involved in the genomic binding site switch of RUNX1 between the LP and Phase 1 stages, we performed a proteomics analysis of stage-specific RUNX1-interacting molecules. Given that RUNX1 overexpression in LPs induces acute cell death, we constructed Myc- and FLAG-tagged RUNX1-ERT2 vectors. LPs and Phase 1 cells were transduced with Myc- and FLAG-tagged RUNX1-ERT2, followed by tamoxifen treatment for 6 h. Tamoxifen-dependent nuclear translocation of Myc- and FLAG-tagged RUNX1-ERT2 was confirmed via immunoblotting (Fig. S2 H). Myc- and FLAG-tagged RUNX1-ERT2–transduced LPs and Phase 1 cells were subjected to two-step affinity purification, followed by SDS-PAGE and silver staining (Fig. 2 C). Liquid chromatography and tandem mass spectrometry (LC-MS/MS) identified RUNX1-interacting molecules in both LPs and Phase 1 cells, including previously reported RUNX1-interacting proteins, such as Cbfβ, p300, and TLE3 (Fig. 2 D, Fig. S2 I, and Table S1) (Mevel et al., 2019; Seo and Taniuchi, 2020). LC-MS/MS results showed that the strong interaction between RUNX1 and CTCF in LPs was considerably attenuated in Phase 1. In contrast, the association of RUNX1 with the Notch1-IC was detected from Phase 1 to Phase 2 (Fig. 2 D and Fig. S2 I). These interactions between RUNX1 and CTCF or Notch1-IC were validated by co-immunoprecipitation (Co-IP) and immunoblotting in LPs and Phase 1 cells (Fig. 2 E). CTCF functions as an insulator, acting concomitantly with cohesin, which consists of four subunits: Smc1a, Smc3, Stag1, and Rad21 (Ong and Corces, 2014). Cohesin also contributes to super-enhancer formation, together with Mediator complexes and p300 (Ramasamy et al., 2023). Spectrometry revealed that interactions between RUNX1 and cohesin subunits, p300, and Mediators, such as Med12, Med13, Med14, and Med17, remained relatively stable between LP and Phase 1, both before and after Notch stimulation (Fig. 2 D and Fig. S2 I). Moreover, RUNX1-Med12 interactions, in which Med12 showed the highest enrichment score among the Mediator components, were comparable between LP and Phase 1, as confirmed by Co-IP (Fig. 2 E). These results indicate that the switching of RUNX1/CTCF to the RUNX1/Notch-IC complex occurs within 2 days following Notch stimulation.

### RUNX regulates a subset of T-signature genes bidirectionally before and after Notch stimulation

**Figure 3.**
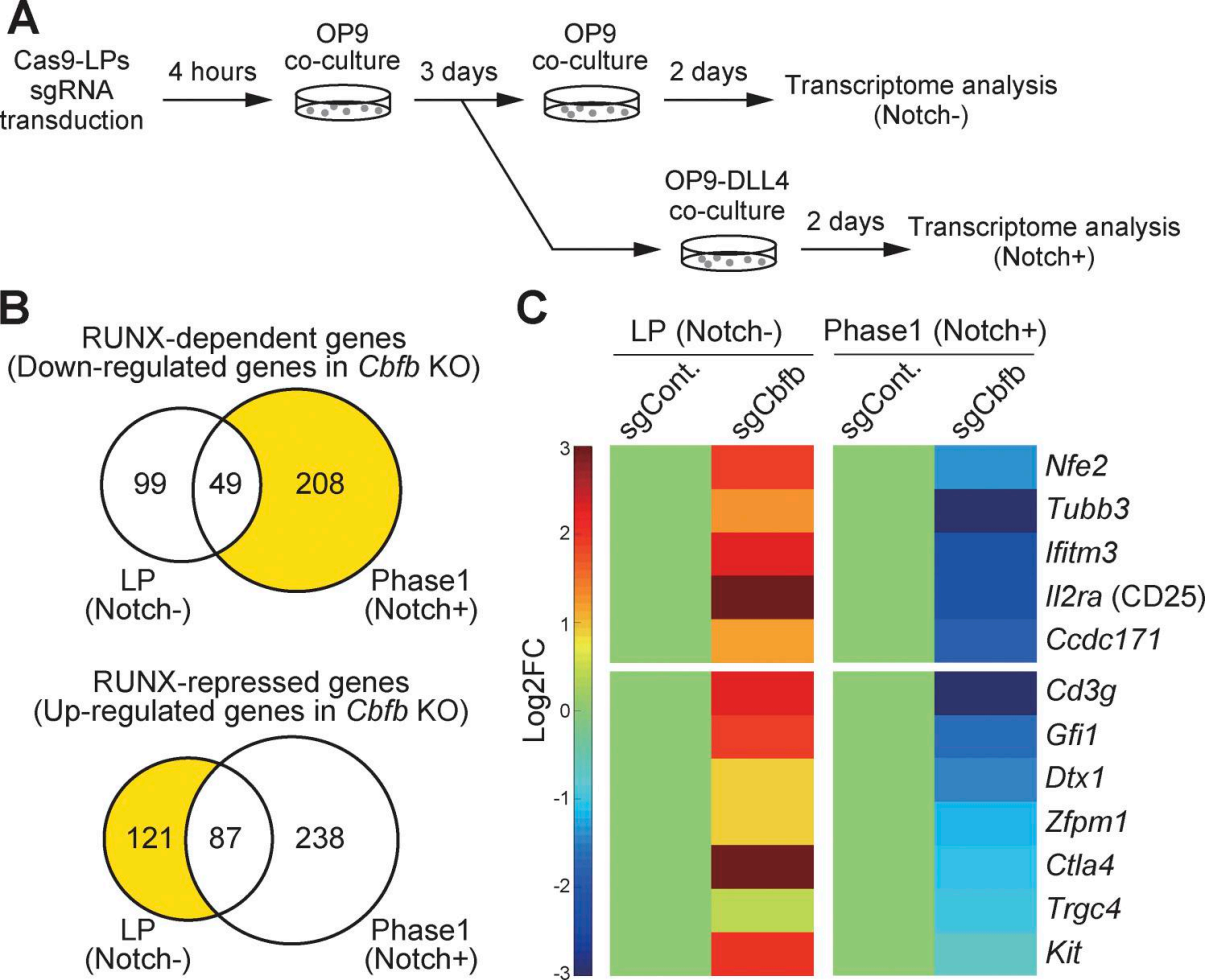
**RUNX represses T-signature genes in LPs and activates them in Phase 1. (A)** Schematic representation of the transcriptome analysis experiment. Retroviral vectors encoding sgRNAs targeting luciferase (sgControl) or *Cbfb* (sgCbfb) were introduced into Cas9-LPs. 5 days after sgRNA introduction, hNGFR^+^ CD45^+^ LP cells were sorted and subjected to QuantSeq 3′ mRNA sequencing. **(B)** Venn diagrams show the numbers of RUNX-dependent and RUNX-repressed DEGs in LPs (Notch-) and Notch-stimulated LPs for 2 days (Phase 1; Notch+). (The full list of the DEGs is provided in Table S2.) **(C)** Heatmaps show the expression changes of representative Notch-activated T-signature genes (Romero-Wolf et al., 2020) associated with T cell development following *Cbfb* deletion. Data are based on the average of three biological replicates.

Samples across our results thus far suggest that RUNX1 forms a complex with CTCF and binds to the CTCF motif during the LP stage. On Notch stimulation, RUNX1 dissociates from CTCF and is redirected to other genomic regions. To further examine the stage-specific roles of RUNX factors in gene regulation, we examined their effects on target gene expression. *Runx1*, *Runx2*, and *Runx3* are co-expressed and function collaboratively in the LP and Phase 1 stages (Shin et al., 2021; Yoshida et al., 2019). To disrupt RUNX TF function, we deleted the *Cbfb* gene, an essential interacting partner for DNA binding among all three RUNX family members. Cas9-LPs were infected with bicistronic retroviral vectors carrying single guide RNAs (sgRNAs) targeting luciferase (control) or *Cbfb* along with a human nerve growth factor receptor (hNGFR) marker. Specific loss of the CBFβ protein was confirmed by immunoblotting, 3 days after sgRNA introduction (Fig. S3 A). To identify stage-specific RUNX target genes in LP and Phase 1 cells, we sorted CD45^+^hNGFR^+^ LPs and Phase 1 cells for transcriptome analysis using QuantSeq 3′ mRNA sequencing 5 days after sgRNA transduction (Fig. 3 A and Fig. S3 B). Differentially expressed genes (DEGs) affected by the deletion of *Cbfb* were defined by adjusted P value <0.05, |Log_2_ fold change (FC)| >1, and average transcripts per kilobase million (TPM) > 10 in either the control or *Cbfb* knockout triplicate experiments (Table S2). The results demonstrated that both RUNX-dependent genes (downregulated in *Cbfb*-deficient cells) and RUNX-repressed genes (upregulated in *Cbfb*-deficient cells) displayed high developmental stage specificity (Fig. 3 B). Most of the RUNX-regulated DEGs were activated or repressed by RUNX factors regardless of developmental stages (Fig. S3 C). However, few of them were regulated in the opposite direction by RUNX in the LP and Phase 1 stages. Indeed, RUNX switched its roles as an activator to a repressor for three genes, i.e., the stem/progenitor gene, *Bcl11a*, between the LP and Phase 1 (Fig. 3 B; and Fig. S3, D and E). Notably, five genes were identified as RUNX-repressed in LP but became RUNX-dependent in Phase 1, among which four genes (*Nfe2*, *Tubb3*, *Ifitm3*, and *Il2ra*) were also recognized as Notch-regulated T-signature genes (Fig. 3 B, yellow area; Fig. S3, D and F) (Romero-Wolf et al., 2020). A comprehensive transcriptome data analysis further revealed that several other Notch-regulated T-signature genes, including *Cd3g*, *Gfil*, *Dtx1*, *Zfpm1*, *Ctla4*, *Trgc4*, and *Kit*, were derepressed in LP but subsequently downregulated in Phase 1 following disruption of *Cbfb* (Fig. 3 C). These findings demonstrate that the RUNX/CBFβ complex regulates a subset of Notch-regulated T-signature genes in a bidirectional manner, acting as a repressor in LP and an activator in Phase 1.

### CTCF represses T-lineage program in LP, and Med12 drives DN2 generation in Phase 1

**Figure 4.**
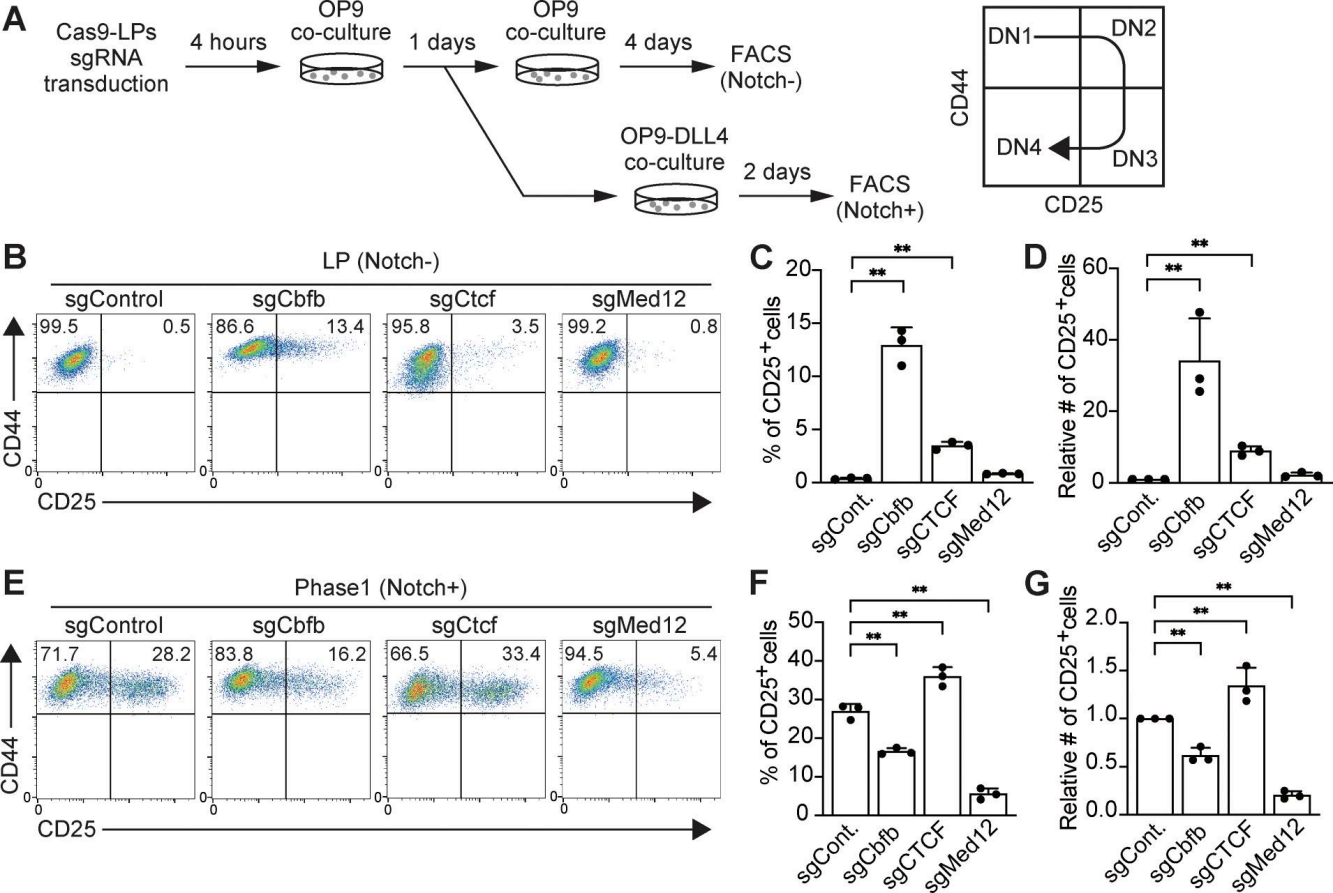
**RUNX/CTCF represses CD25** ^**+**^ **cell generation in LPs, whereas RUNX/Mediator promotes DN2 development after Notch stimulation. (A)** Schematic of the stage-specific deletion of *Cbfb*, *Ctcf*, or *Med12* in LP and Phase 1 cells using the CRISPR/Cas9 system. **(B)** Retroviral vectors encoding sgRNAs were introduced into Cas9-LPs. 5 days after sgRNA introduction, hNGFR^+^CD45^+^ sgRNA-transduced cells were gated and analyzed for CD44 and CD25 expression. **(C and D)** Percentage (C) and relative number (D) of CD25^+^ cells among hNGFR^+^CD45^+^ sgRNA-transduced cells from B are shown with SD. **(E)** 1 day after sgRNA introduction, LPs were transferred onto OP9-DLL4 stromal cells and cocultured for 2 days. hNGFR^+^CD45^+^ sgRNA-transduced cells were gated and analyzed for CD44 and CD25 expression. **(F and G)** Percentage (F) and relative number (G) of CD25^+^ cells among hNGFR^+^CD45^+^ sgRNA-transduced cells from E are shown with SD. Data in B and E are representative of three independent experiments. Data in C, D, F, and G represent mean values from three independent biological replicates. The data were analyzed by one-way ANOVA with Dunnett’s multiple comparisons (C, D, F, and G). For LP (Notch-); C, **adjusted P < 0.0001 for sgCbfb; **adjusted P = 0.0064 for sgCTCF. For LP (Notch-); D, **adjusted P < 0.0001 for sgCbfb; **adjusted P = 0.0064 for sgCTCF. For Phase 1 (Notch+); F, **adjusted P = 0.0046 for sgCbfb; **adjusted P = 0.0002 for sgCTCF; **adjusted P = 0.0362 for sgMed12. For Phase 1 (Notch+); G, **adjusted P = 0.0049 for sgCbfb; **adjusted P = 0.0002 for sgCTCF; **adjusted P = 0.0360 for sgMed12. SD, standard deviation.

Next, we explored the roles of RUNX1-interacting molecules in T-lineage initiation. For this, we examined CTCF, a RUNX1-interacting partner specifically in LP, and Med12, a consistent RUNX1-interacting molecule and a major subunit of the Mediator complex. sgRNAs targeting *Cbfb*, *Ctcf*, or *Med12* were transduced into Cas9-LPs; T-progenitor development was assessed using the markers CD44 and CD25 in CD45^+^hNGFR^+^ cells (Fig. 4 A and Fig. S3 A). The deletion of *Cbfb* or *Ctcf* induced the spontaneous generation of CD25^+^ DN2-like cells without Notch stimulation (Fig. 4, B–D). In contrast, disruption of *Cbfb* or *Med12* in Phase 1 cells severely impaired their progression to the DN2 stage following Notch stimulation (Fig. 4, E–G). These results suggest that the RUNX/CTCF transcriptional repressive complex actively suppresses the spontaneous activation of the T-lineage program in LPs, whereas the RUNX/Mediator transcriptional activation complex is essential for driving T cell development after Notch stimulation in Phase 1.

### Stage-specific RUNX1 binding: Genomic regions co-occupied with CTCF or Med12

We hypothesized that the RUNX1/CTCF transcriptional repressive complex suppresses T-signature genes in LPs, whereas the RUNX1/Mediator transcriptional activation complex enhances T-signature gene expression in Phase 1. To verify this model, we performed ChIP-seq analyses for CTCF and Med12 in LPs and Phase 1 cells, comparing them with stage-specific RUNX1 genomic occupancy.

**Figure 5.**
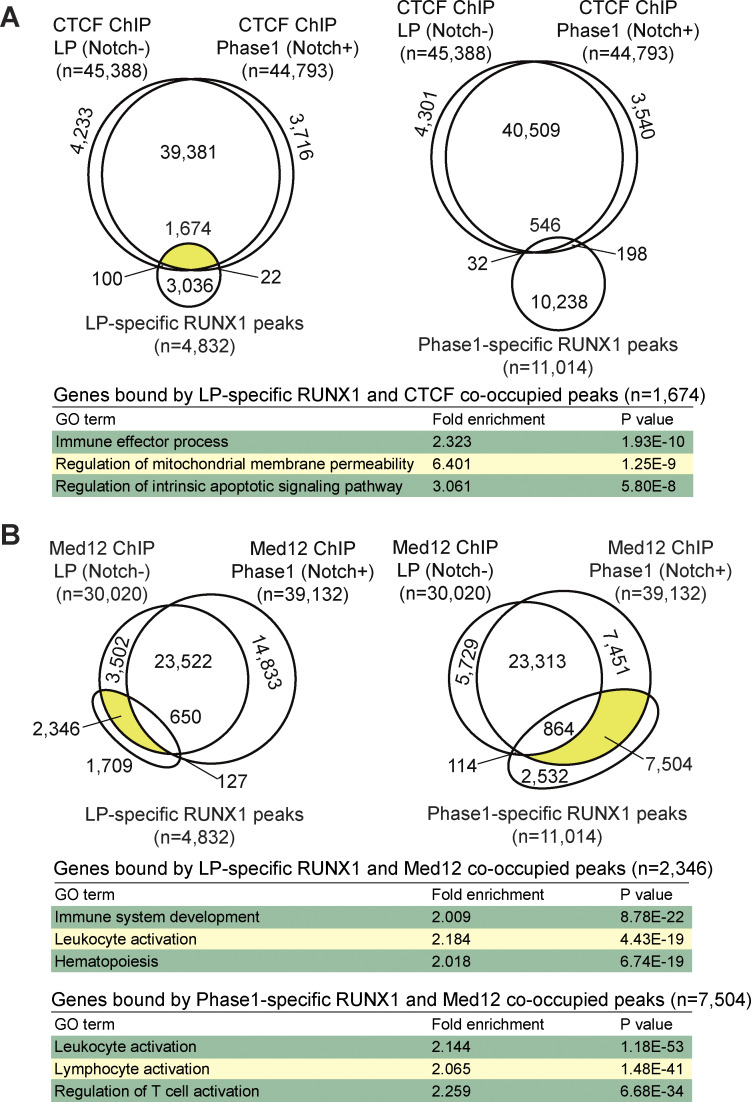
**RUNX1/CTCF represses T-signature genes in LPs, whereas RUNX1/Med12 activates them in Phase 1 cells. (A)** ChIP-seq analyses for RUNX1, CTCF, and Med12 were performed using Cas9-LPs with or without Notch stimulation. Venn diagrams show the numbers of reproducible CTCF ChIP peaks overlapping with LP-specific (left) or Phase 1–specific (right) RUNX1 peaks (as shown in Fig. 2 A). Genes bound by CTCF at LP-specific RUNX1 peaks (highlighted in yellow) were subjected to GO analysis using the GREAT analysis tool (https://great.stanford.edu/public/html/). The top three GO terms are shown. **(B)** Venn diagrams show the numbers of reproducible Med12 ChIP peaks overlapping with LP-specific (left) or Phase 1–specific (right) RUNX1 peaks (as shown in Fig. 2 A). ChIP peaks co-occupied by LP-specific RUNX1 and Med12, as well as Phase 1–specific RUNX1 and Med12, are highlighted in yellow. The top three GO terms for genes bound by LP-specific RUNX1 and LP-specific Med12-co-occupied peaks (upper), and genes bound by Phase 1-specific RUNX1 and Phase 1-specific Med12 (lower) are shown. Data were obtained from ChIP-seq peaks scored as reproducible in two replicate samples.

**Figure S4.**
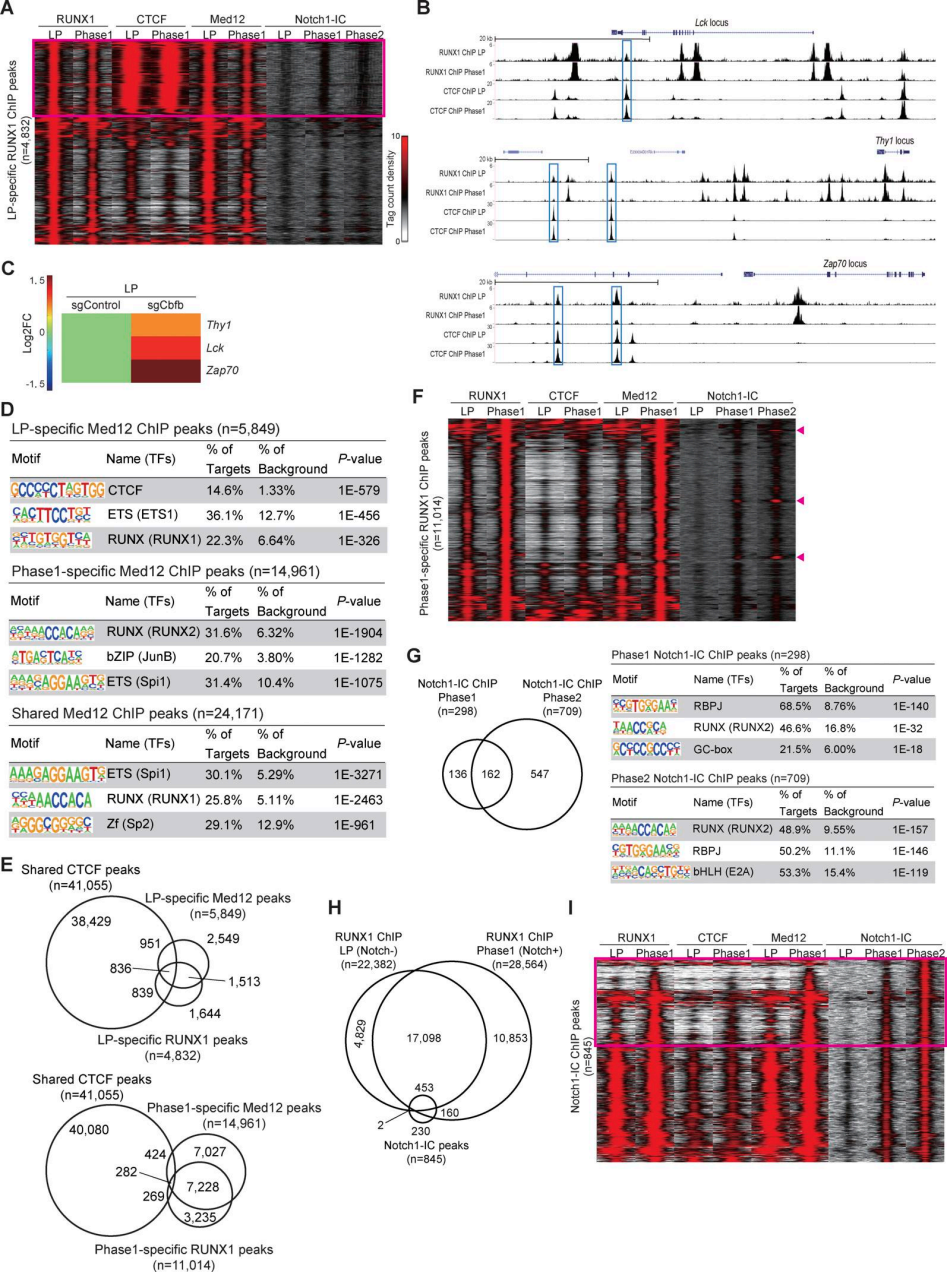
**ChIP-seq analysis of RUNX1, CTCF, Med12, and Notch1-IC binding in LP and Phase 1 cells. (A)** Tag count distributions for RUNX1, CTCF, Med12, and Notch1-IC ChIP peaks around LP-specific RUNX1-binding regions are shown as peak-centered heatmaps. Each lane represents the merged tag directories from two biological replicates. **(B)** Representative ChIP-seq tracks for RUNX1 and CTCF in LP and Phase 1 around the *Lck*, *Thy1*, and *Zap70* loci. CTCF-binding sites co-occupied with LP-specific RUNX1 peaks are labeled with blue rectangles. Data are representative of two independent experiments. **(C)** Heatmap showing changes in the expression of *Thy1*, *Lck*, and *Zap70* in LP following *Cbfb* deletion. Data are presented as the average of three biological replicates. **(D)** Top three enriched sequence motifs among the 5,849 LP-specific, the 14,961 Phase 1–specific, and 24,171 shared reproducible Med12 peaks between LP and Phase 1 are shown (Fig. 5 B). Data are based on ChIP-seq peaks scored as reproducible in two replicate samples. **(E)** Venn diagrams show the number of shared CTCF ChIP peaks overlapping with LP-specific (upper) or Phase 1–specific (lower) Med12 and RUNX1 peaks. **(F)** Tag count distributions for RUNX1, CTCF, Med12, and Notch1-IC ChIP peaks around Phase 1–specific RUNX1-binding regions are shown as peak-centered heatmaps. Each lane represents the merged tag directories from two biological replicates. **(G)** ChIP-seq data for Notch1-IC in Phase 1 and Phase 2 were analyzed. Venn diagrams show the number of reproducible Notch1-IC ChIP peaks in Phase 1 and Phase 2 cells. The top three enriched sequence motifs among the 298 reproducible Phase 1 Notch1-IC peaks (upper panel) and 709 reproducible Phase 2 Notch1-IC peaks (lower panel) are shown. **(H)** Venn diagrams showing the number of reproducible RUNX1 ChIP peaks in LP and Phase 1 cells, along with Notch1-IC ChIP peaks in Notch-stimulated pro-T cells (Phase 1 + Phase 2, *n* = 845). **(I)** Tag count distributions for RUNX1, CTCF, Med12, and Notch1-IC ChIP peaks around Notch1-IC–binding regions in Notch-stimulated pro-T cells are shown as a peak-centered heatmap. Each lane represents the merged tag directories from two biological replicates.

CTCF ChIP peaks remained relatively stable before and after Notch stimulation; however, certain LP-specific RUNX1 peaks were co-occupied with CTCF-binding sites (Fig. 5 A, left, yellow area), whereas Phase 1–specific RUNX1 peaks showed reduced CTCF co-occupancy (Fig. 5 A, right). Notably, ∼30% of LP-specific RUNX1 overlapped with CTCF peaks without any Notch1-IC–binding signal enrichment (Fig. 5 A and Fig. S4 A, magenta rectangle). Gene Ontology (GO) analysis showed that genes associated with LP-specific RUNX1/CTCF-co-occupied peaks (Fig. 5 A, left, yellow area) were significantly enriched for “immune effector processes,” which included many T-signature genes, such as *Lck*, *Zap70*, and *Thy1*. Indeed, LP-specific RUNX1/CTCF-co-occupied peaks were found around the *Lck*, *Zap70*, and *Thy1* loci, and their expression levels were increased by the deletion of *Cbfb* in LP (Fig. S4, B and C). These results suggest that the RUNX1/CTCF transcriptional repressive complex binds T-signature loci and inhibits their expression in LPs.

In contrast to CTCF, Med12 occupancy exhibited marked site switching across the genome, in line with the dynamic redistribution of RUNX1. Many LP-specific RUNX1 peaks overlapped with LP-specific Med12 peaks, and Phase 1–specific RUNX1 peaks strongly correlated with Phase 1–specific Med12 peaks (Fig. 5 B, yellow areas). As seen in the stage-specific RUNX1 ChIP peaks (Fig. 2 B), motif analysis showed the highest enrichment of the CTCF motif for the LP-specific Med12 peaks and the enriched Spi1 and RUNX motifs near the Phase 1–specific and shared Med12 peaks (Fig. S4 D). Moreover, the overlapping patterns of stage-specific Med12 and RUNX1 peaks with the CTCF-binding genomic regions were similar (Fig. S4 E). GO analysis showed that genes bound by LP-specific RUNX1/Med12-co-occupied peaks (Fig. 5 B, left, yellow area) were enriched for “immune system development” and “hematopoiesis,” whereas the genes linked to Phase 1–specific RUNX1/Med12-co-occupied peaks (Fig. 5 B, right, yellow area) were enriched for “leukocyte activation” and “regulation of T cell activation.” These results suggest that although the RUNX1/Mediator transactivation complex remains functionally consistent, its genomic targets undergo dramatic redirection, shifting from stem cell/progenitor signature genes to T-signature genes on Notch signaling.

A Phase 1–specific RUNX1 peak-centered heatmap showed that Med12 was recruited to Phase 1–specific RUNX1-binding sites, without enrichment of CTCF signals (Fig. S4 F). Importantly, a small subset of Phase 1–specific RUNX1 peaks were directly bound by Notch1-IC (Fig. S4 F, magenta arrowheads). Further analysis revealed that in Phase 1 and Phase 2, the RBPJ (a DNA-binding subunit of the Notch1–IC complex) motif co-enriched at Notch-IC occupancy sites, along with the RUNX motif (Fig. S4 G). However, Notch1-IC ChIP peaks were markedly lower than RUNX1 peaks (Fig. S4 H). Moreover, most Notch1-IC ChIP peaks were cobound by RUNX1/Med12 in Phase 1, with approximately one third of these being Phase 1–specific Notch1-IC–dependent peaks (Fig. S4, H and I, magenta rectangle).

### Notch target genes bound by the LP-specific RUNX1/CTCF and the Phase 1–specific RUNX1/Mediator/p300 complexes

**Figure 6.**
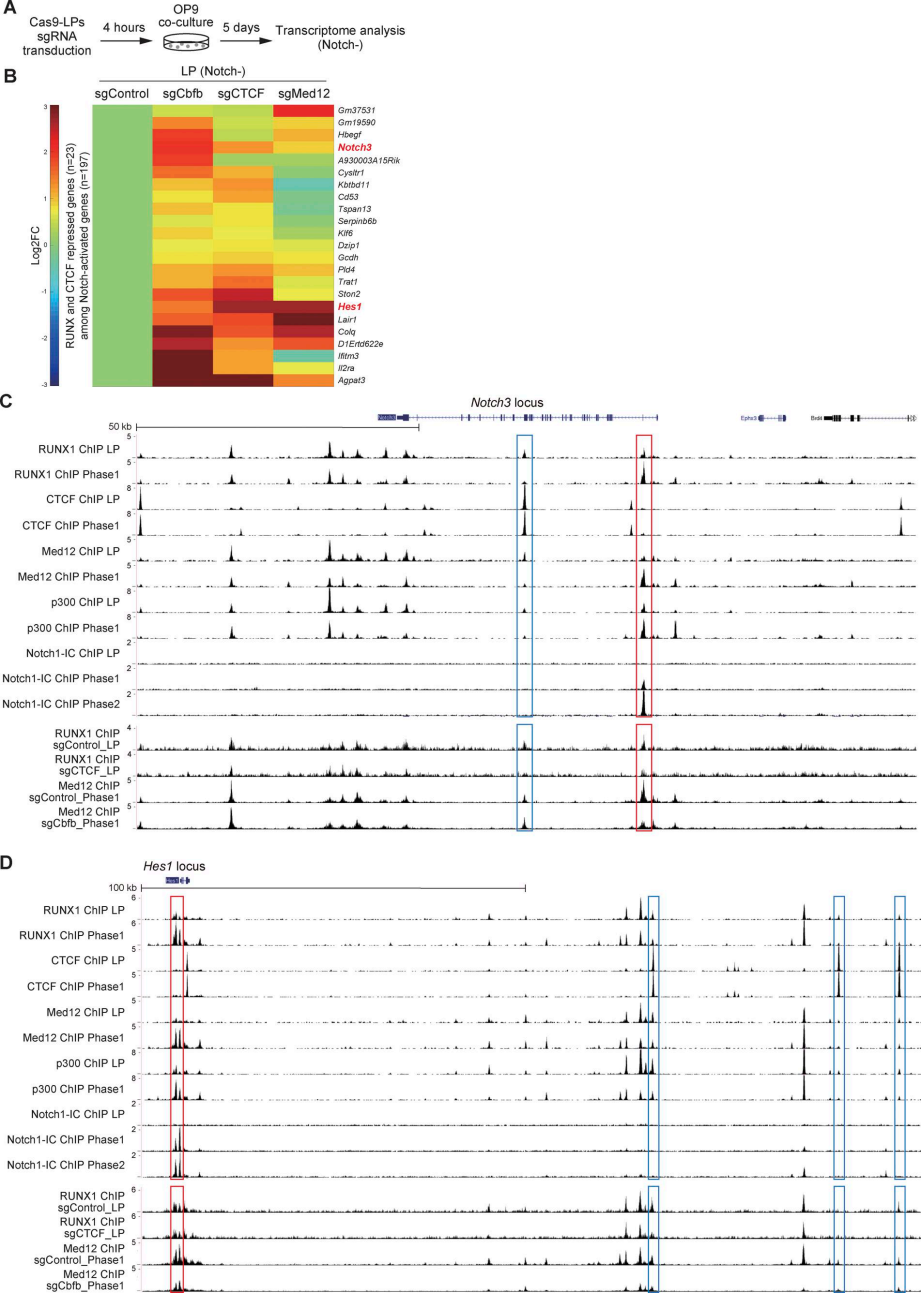
**Organization of LP-specific RUNX1/CTCF repressive complex and Phase 1–specific RUNX1/Med12/p300/Notch1-IC activation complex at the *Notch3* and *Hes1* loci. (A)** Experimental scheme for the transcriptome analysis. **(B)** 5 days after sgRNA introduction, hNGFR^+^CD45^+^ LP cells were sorted, and subjected to QuantSeq 3′ mRNA sequencing. The heatmap shows the expression changes of representative RUNX1- and CTCF-repressed genes among Notch-activated genes in pro-T cell stages (Romero-Wolf et al., 2020). Data represent the average of three biological replicates. **(C and D)** Representative ChIP-seq tracks for RUNX1, CTCF, Med12, and p300 in LP and Phase 1 cells, Notch1-IC in LP, Phase 1, and Phase 2 cells, RUNX1 in sgControl- or sgCTCF-transduced LPs, and Med12 in sgControl- or sgCbfb-transduced Phase 1 cells around the *Notch3* (C) and *Hes1* (D) loci are shown. CTCF-binding sites co-occupied with LP-specific RUNX1 peaks are labeled with blue rectangles, whereas Phase 1–specific RUNX1/Med12/p300/Notch-IC–binding sites are marked with red rectangles. Data are representative of two independent experiments.

**Figure S5.**
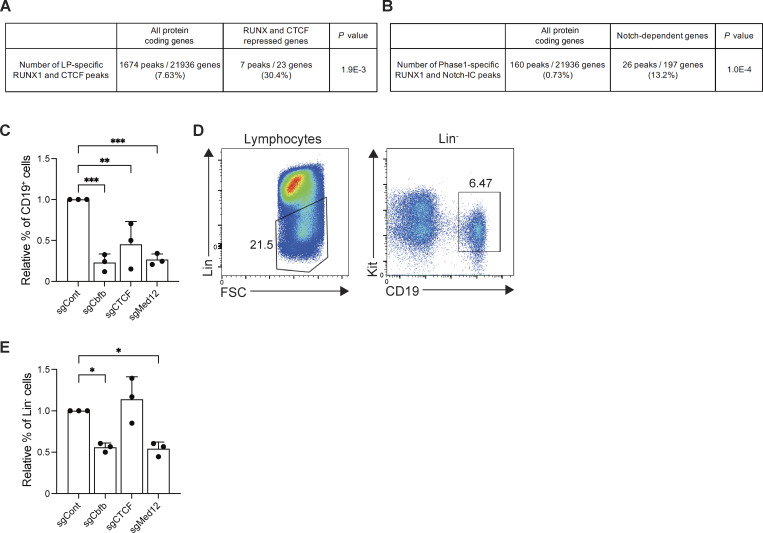
**Notch-dependent functional conversion of RUNX TFs in T-lineage commitment. (A)** Number of LP-specific RUNX1 peaks co-occupied with CTCF in whole genome or RUNX- and CTCF-repressed among Notch-activated genes (Fig. 6 B) are shown. The data were analyzed using a chi-square test with Yates’s correction. **(B)** Number of Phase 1–specific RUNX1 peaks co-occupied with Notch1-IC in whole genome or Notch-activated genes are shown. The data were analyzed using a chi-square test with Yates’s correction. **(C)** Relative percentage of CD19^+^ cells among CD45^+^Lin^−^ cells from Fig. 8 A are shown, including their SDs. **(D)** BM progenitors were cocultured with OP9, and the B cell developmental status was scored using the markers CD19 and Kit among CD45^+^Lin^-^ cells on day 4. **(E)** Relative percentage of Lin^−^CD19^−^ cells among CD45^+^ cells from Fig. 8 D are shown, including their SDs. Data in C and E represent mean values from three independent biological replicates. Data in D are representative of two independent experiments. The data were analyzed using a one-way ANOVA with Dunnett’s multiple comparisons (C and E). For C: CD19^+^, ***adjusted P = 0.0007 for sgCbfb; **adjusted P = 0.006 for sgCTCF; ***adjusted P = 0.001 for sgMed12. For E: Lin^−^, *adjusted P = 0.0145 for sgCbfb; *adjusted P = 0.0116 for sgMed12. SD, standard deviation.

Our results indicate that the RUNX1/CTCF complex represses T-signature genes, whereas the RUNX1/Mediator complex activates stem/progenitor-related genes in LPs. On Notch activation, the RUNX1/CTCF complex reorganizes into the RUNX1/Notch-IC complex and the RUNX1/Mediator complex is redirected to T-signature genes. To identify specific Notch target genes regulated by this mechanism, we subjected *Cbfb*-, *Ctcf*-, or *Med12*-deficient LPs to transcriptome analysis (Fig. 6 A). In addition to the generation of CD25^+^ cells without Notch stimulation (Fig. 4, A–D), the deletion of *Cbfb* or *Ctcf* induced the expression of several Notch-activated genes, including functionally important T-signature genes *Notch3* and Hairy/Enhancer of Split 1(*Hes1*) (Fig. 6 B). ChIP-seq revealed LP-specific RUNX1/CTCF binding and Phase 1–specific Notch1-IC/RUNX1/Med12/p300 occupancy at the *Notch3* and *Hes1* loci. At the *Notch3* locus, a consistent CTCF peak was detected at intron 24, along with weak but reproducible LP-specific RUNX1 binding (Fig. 6 C, blue rectangle). At the *Hes1* locus, no CTCF peaks were observed within the gene body, but three peaks were detected more than +100 kb downstream. These sites exhibited faint but reproducible LP-specific RUNX1 binding (Fig. 6 D, blue rectangles). Importantly, these LP-specific RUNX1 peaks around the *Notch3* and *Hes1* loci were regulated by CTCF because their binding signal was weakened by the deletion of CTCF in LP (Fig. 6, C and D, blue rectangles). The genome contains 21,936 protein-coding genes with 1,674 LP-specific RUNX1 peaks co-occupied with CTCF (Fig. 5 A, left, yellow area). In contrast, 7 LP-specific RUNX1/CTCF-co-occupied peaks were detected around the 23 Notch target genes whose expression increased upon the deletion of *Cbfb* and *Ctcf* in LP, showing significant enrichment (Fig. 6 B and Fig. S5 A).

**Figure 7.**
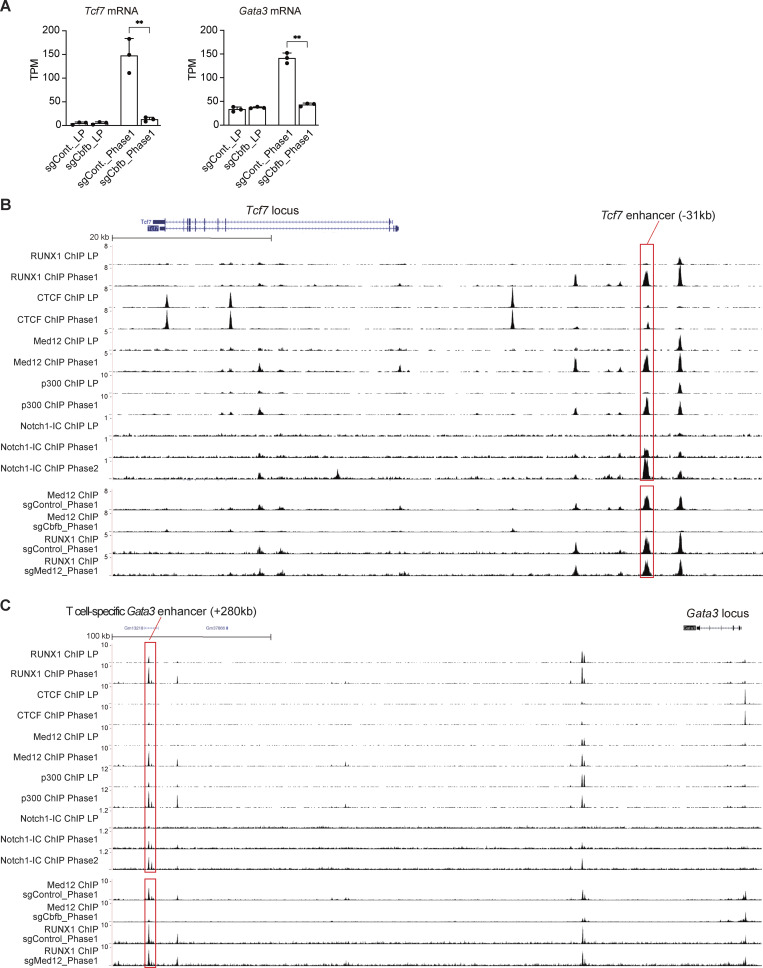
**RUNX1, CTCF, Med12, p300, and Notch1-IC–binding at the *Tcf7* and *Gata3* loci in LP and Phase 1 cells. (A)** TPM values for *Tcf7* and *Gata3* in *Cbfb*-deficient LPs or Phase 1 cells are shown with SDs. The data represent the mean values of three independent biological replicates. The data were analyzed by a two-sided *t* test. For *Tcf7* mRNA, **P = 0.003. For *GATA3* mRNA, **P = 0.0001. **(B and C)** Representative ChIP-seq tracks for RUNX1, CTCF, Med12, and p300 in LP and Phase 1, Notch1-IC in LP, Phase 1, and Phase 2, Med12 in sgControl- or sgCbfb-transduced Phase 1 cells, and RUNX1 in sgControl- or sgMed12-transduced Phase 1 cells around the *Tcf7* (B) and *Gata3* (C) loci. Phase 1–specific RUNX1/Med12/p300/Notch-IC–binding sites are labeled with red rectangles, including the *Tcf7* enhancer (−31 kb) and T cell–specific *Gata3* enhancer (+280 kb) regions. Data are representative of two independent experiments. SD, standard deviation.

Following Notch stimulation, Notch1-IC directly bound to the intron 1 region of the *Notch3* locus, where the modest RUNX1-binding signal detected at the Notch1-IC–binding site in the LPs was clearly enhanced in Phase 1 with the corecruitment of Med12 and p300 (Fig. 6 C, red rectangle). Moreover, Notch-IC–mediated recruitment of the RUNX1/Med12/p300 transcriptional activation complex was observed around the transcriptional start site of *Hes1* (Fig. 6 D, red rectangle). Expression levels of *Tcf7* and *Gata3*, two early and functionally critical Notch target genes, were significantly decreased in *Cbfb*-deficient Phase 1 cells (Fig. 7 A and Table S2). Notably, the *Tcf7* enhancer (−31 kb) (Harly et al., 2020; Weber et al., 2011) and the T cell–specific *Gata3* enhancer (+280 kb) (Hosoya-Ohmura et al., 2011; Ohmura et al., 2016) were directly bound by Notch1-IC along with the RUNX1/Med12/p300 complex, particularly after Notch stimulation (Fig. 7, B and C, red rectangles). The recruitment of Med12 at the Notch1-IC–binding regions around the *Notch3*, *Hes1*, *Tcf7*, and *Gata3* loci was RUNX-dependent. Med12 ChIP peaks detected at the Notch1-IC–binding sites in Phase 1 were attenuated by the disruption of *Cbfb* (Fig. 6, C and D; and Fig. 7, B and C, red rectangles), but RUNX1 peaks were not affected by the deletion of Med12 (Fig. 7, B and C, red rectangles). There are 160 Notch1-IC peaks co-occupied with Phase 1–specific RUNX1 peaks across the genome with 21,936 protein-coding genes (Fig. S4 H). In contrast, 26 Phase 1–specific RUNX1/Notch1-IC peaks were found around the 197 Notch-dependent genes, showing significant enrichment (Fig. 6 B and Fig. S5 B). Therefore, to trigger the T-lineage transcriptional program, Notch1-IC not only derepresses T-signature gene expression via switching from the RUNX1/CTCF complex to RUNX1/Notch1-IC but also redirects the RUNX1/Mediator/p300 transcriptional activation complex to regulatory elements of its target loci.

### RUNX, CTCF, and Med12 regulate the initiation of T-lineage program in primary BM progenitors

**Figure 8.**
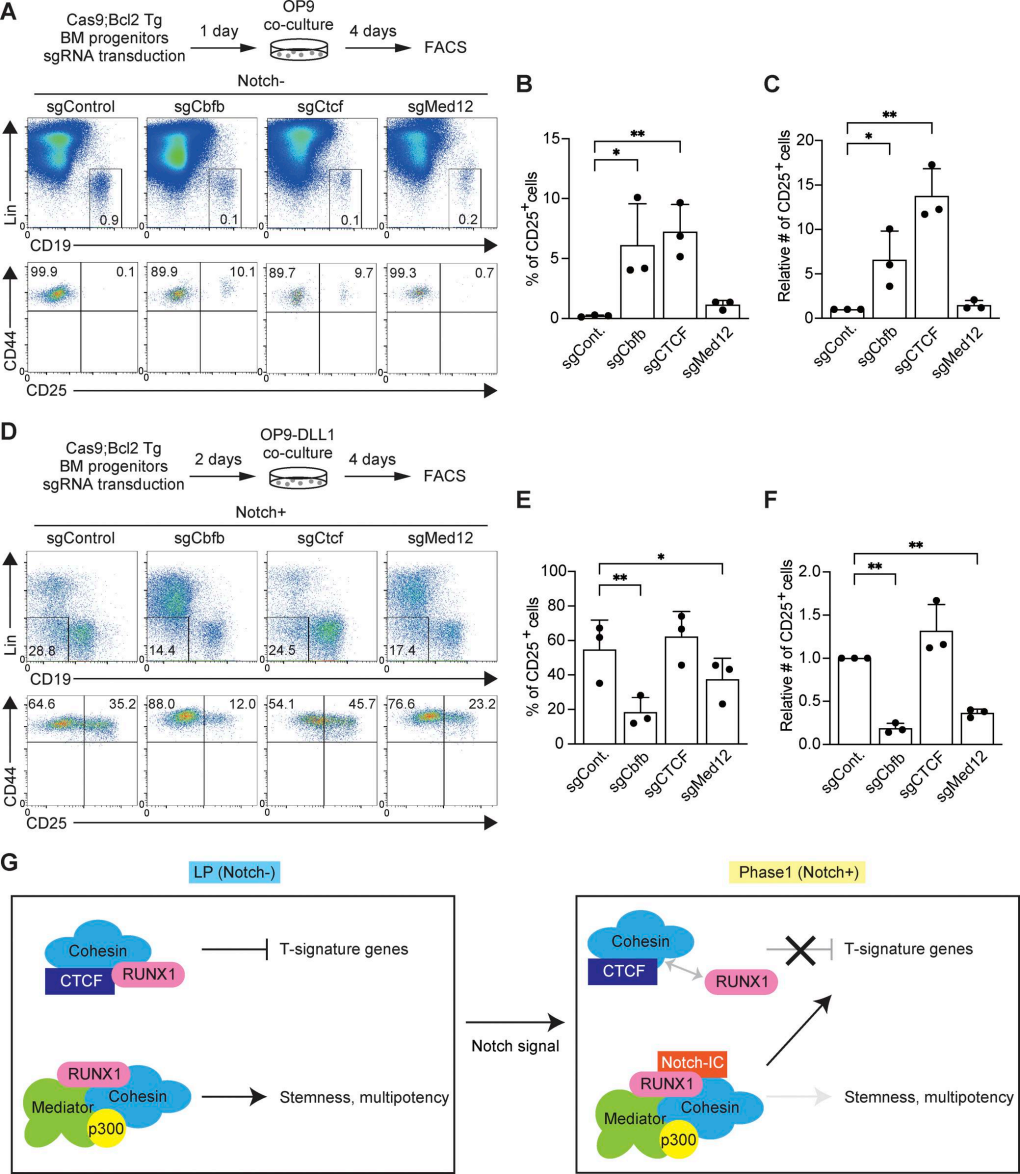
**Effects of RUNX, CTCF, or Med12 deletion on DN2 cell generation before and after Notch stimulation in primary BM progenitors. (A)** Experimental scheme. BM progenitors from Cas9;Bcl2 Tg mice were transduced with sgRNA and cultured without stromal cells for 1 day. Then, they were transferred onto OP9 stromal cells and cocultured for 4 days. CD45^+^hNGFR^+^ sgRNA-transduced cells were gated and analyzed for Lin markers, CD19, CD44, and CD25 expression. Representative plots show Lin/CD19 profiles in CD45^+^hNGFR^+^ sgRNA-transduced cells (upper panel) and CD44/CD25 profiles in CD45^+^hNGFR^+^Lin^−^CD19^+^ cells (lower panel). **(B and C)** Percentage (B) and relative number (C) of CD25^+^ cells among CD45^+^hNGFR^+^Lin^−^CD19^+^ sgRNA-transduced cells (from A) are shown with SD. **(D)** Alternative experimental scheme. sgRNA-transduced BM progenitors were cultured without stromal cells for 2 days. Then, they were transferred onto OP9-DLL1 stromal cells and cocultured for 4 days. CD45^+^hNGFR^+^ sgRNA-transduced cells were gated and analyzed for Lin markers, CD19, CD44, and CD25 expression. Representative plots show Lin/CD19 profiles in CD45^+^hNGFR^+^ sgRNA-transduced cells (upper panel) and CD44/CD25 profiles in CD45^+^hNGFR^+^Lin^−^CD19^−^ cells (lower panel). **(E and F)** Percentage and relative number of CD25^+^ cells among hNGFR^+^CD45^+^Lin^−^CD19^−^ sgRNA-transduced cells (from D) are shown with SD. **(G)** Working model of Notch-dependent functional conversion of RUNX TFs regulating the initiation of the T-lineage program. The RUNX/CTCF complex in the LP stage represses T-signature genes. Notch signaling induces the dissociation of RUNX from CTCF and facilitates the redirection of the RUNX/Mediator/p300 complex, thereby triggering T cell differentiation. Results shown in A and D are representative of three independent experiments, whereas data in B, C, E, and F represent the mean values of three independent biological replicates. The data were analyzed by one-way ANOVA with Dunnett’s multiple comparisons (B, C, E, and F). For Notch-: B, *adjusted P = 0.0224 for sgCbfb; **adjusted P < 0.0001 for sgCTCF. For Notch-: C, *adjusted P = 0.0208 for sgCbfb; **adjusted P = 0.0083 for sgCTCF. For Notch+; E, **adjusted P = 0.0014 for sgCbfb; *adjusted P = 0.0337 for sgMed12. For Notch+: F, **adjusted P = 0.0006 for sgCbfb; **adjusted P = 0.0029 for sgMed12. SD, standard deviation.

Finally, to validate that RUNX1/CTCF complexes serve to restrain T-lineage specific genes in progenitor cells, we used primary BM-derived hematopoietic progenitors from Rosa26-Cas9 knock-in mice, which carry a *Bcl2* transgene (*Cas9;Bcl2* Tg). This transgene enhances viable recovery without affecting T cell development (Yui et al., 2010). Progenitors were transduced with sgRNA targeting *Cbfb*, *Ctcf*, or *Med12* and cultured without OP9 stromal cells for 1–2 days. The cells were then transferred onto OP9 or OP9-Delta-like 1 (DLL1) monolayers to induce Notch signaling. After 4 days of coculture, lineage markers (Lin), CD45, CD19, CD44, and CD25, were analyzed to determine T cell developmental stages. In the absence of Notch signaling (OP9 coculture, which supports B cell differentiation), the decreased generation of CD19^+^ B progenitors was confirmed through the deletion of *Cbfb*, *Ctcf*, or *Med12*. Therefore, these three factors positively contribute to the early B cell development (Fig. S5 C). It is known that one of the pro-T cell markers CD25 is also expressed in the CD19^+^Kit^−^pre-BCR^+^ pre-B cell stage. CD19^+^ B progenitors detected on day 4 under our B cell culture conditions had intermediate Kit expression; thus, they would be pre-pro-B or pro-B stages (Fig. S5 D). However, *Cbfb* or *Ctcf* disruption led to the generation of abnormal CD44^+^CD25^+^ cells from CD45^+^Lin^−^CD19^+^ B progenitors (Fig. 8, A–C). In contrast, under Notch stimulation (OP9-DLL1 coculture), disruption of *Cbfb* or *Med12* significantly reduced both the percentage and number of CD44^+^CD25^+^ DN2 cells among CD45^+^Lin^−^CD19^−^ progenitors (Fig. 8, D–F; and Fig. S5 E). Thus, consistent with findings in Cas9-LP cell lines, RUNX and CTCF suppress the generation of CD25^+^ cells in the absence of Notch signal, whereas RUNX and Med12 are essential for T cell differentiation in Notch-stimulated Phase 1 cells derived from primary hematopoietic progenitors.

## Discussion

RUNX TFs are crucial for the development of multiple hematopoietic lineages, including T cells, via cellular context– and developmental stage–specific functions (de Bruijn and Dzierzak, 2017; Hosokawa et al., 2021a; Mevel et al., 2019; Seo and Taniuchi, 2020; Shin et al., 2021). We recently demonstrated that RUNX-binding genomic regions and their target genes undergo dynamic shifts during the transition from Phase 1 to Phase 2, specifically at the T-lineage commitment checkpoint (Hosokawa et al., 2021a; Shin et al., 2021). In Phase 1, RUNX1 maintains multipotency in collaboration with PU.1, whereas in Phase 2, it partners with Bcl11b to drive T-lineage specification. Thus, RUNX1 undergoes dynamic partner switching, resulting in its redeployment at the T-lineage commitment checkpoint. In this study, we attempted to reveal the functional dynamics of RUNX factors between the LP and the Notch-stimulated T progenitor stage (Phase 1). Using a biochemical approach with highly tractable and physiologically relevant Cas9-LP cell lines (Fig. 1), we identified CTCF as an LP-specific RUNX1-interacting partner (Fig. 2). Consistently, LP-specific RUNX1-binding genomic sites were significantly enriched for the CTCF consensus motif, and many were co-occupied by CTCF (Fig. 2, Fig. 5, and Fig. S4 A). Following Notch stimulation, Notch1-IC directly interacted with RUNX1 and recruited it to Notch-regulated T-signature gene loci (Figs. 2, 6, 7, and S4). These findings suggest that the transition from RUNX1/CTCF to RUNX1/Notch1-IC complexes marks the initiation of the Notch-mediated T-lineage program. However, the mechanism by which RUNX1 dissociates from CTCF on Notch stimulation remains unknown. One possibility is that Notch1-IC competes with CTCF for RUNX1 binding, reminiscent of the competitive interaction between PU.1 and Bcl11b at the T-lineage commitment checkpoint (Hosokawa et al., 2018a; Hosokawa et al., 2018b; Shin et al., 2021). Another possibility is that Notch signaling alters the posttranslational modification status of RUNX1 and/or CTCF, thereby influencing their interactions. Posttranslational modifications, including phosphorylation, acetylation, methylation, glycosylation, and SUMOylation, are well known to regulate the functional complexity of both RUNX factors and CTCF (Del Rosario et al., 2019; Guo and Friedman, 2011; Jin et al., 2004; Kim et al., 2014; Kitchen and Schoenherr, 2010; Luo et al., 2020; Tang et al., 2024; Zhao et al., 2008).

Notch1-IC directly recruits the RUNX1/Med12/p300 transcriptional activation complex to key target loci such as *Tcf7*, *Gata3*, *Notch3*, and *Hes1* (Fig. 6, C and D; and Fig. 7, B and C). The number of Notch1-IC peaks in pro-T cell stages was significantly lower than that of RUNX1 peaks. Approximately 800 Notch1-IC peaks were detected in pro-T cells, whereas >20,000 RUNX1 peaks were observed in Phase 1 (Fig. 2 A; and Fig. S4, G and H). As for Phase 1–specific RUNX1, the number of peaks co-occupied by Phase 1–specific Med12 peaks was ∼7,500 (Fig. 5 B). This suggests that many Notch-dependent redirections of the RUNX1/Med12 transactivation complex occur through indirect regulation (Fig. S4, F and H). Among the earliest direct Notch target genes are *Tcf7* and *Gata3*, which encode TFs crucial for T-lineage specification (Hosokawa and Rothenberg, 2021; Scripture-Adams et al., 2014; Shin et al., 2024; Weber et al., 2011). In collaboration with Notch1-IC, TCF1 (encoded by *Tcf7*) and GATA3 activate various T-signature genes (Hosokawa and Rothenberg, 2021; Shin et al., 2024). In addition, TCF1 functions as a pioneer factor in T cell development (Johnson et al., 2018). Thus, Notch1-IC directly activates a relatively small set of functionally important T-signature genes, whereas these direct Notch targets, including TCF1 and GATA3, likely cooperate to promote T-lineage specification by redirecting the RUNX1/Med12/p300 complex.

Disruption of RUNX factors in LPs led to the derepression of some T-signature genes, similar to the effect observed on *Ctcf* deletion (Fig. 4 B and Fig. 6 B). These results suggest that RUNX factors contribute to CTCF-mediated repression of spontaneous T-signature gene activation in LPs. CTCF is a well-known architectural protein that regulates gene repression, activation, and formation of higher order chromatin structures (Ong and Corces, 2014; Phillips and Corces, 2009). However, the molecular mechanisms underlying its context-dependent roles remain unclear. In LPs, CTCF appears to act as a repressor of *Hes1* and *Notch3*, two of functionally important Notch-activated T-signature genes. Because CTCF binding at the T-signature loci remains consistent before and after Notch stimulation, its repressor activity may be regulated through its complexation with RUNX1 (Fig. 6, C and D). Stage-specific interactions of CTCF with TFs provide mechanistic explanations for its context-dependent regulatory functions.

RUNX factors bind to their consensus DNA motif to regulate gene expression. However, LP-specific RUNX1-binding genomic regions showed the highest enrichment in the CTCF motif (Fig. 2 B). This suggests that many RUNX1-binding sites in LPs are determined by CTCF. Among the Phase 1–specific RUNX1-binding regions, PU.1, encoded by the *Spi1* gene, was the second most enriched motif (Fig. 2 B). Indeed, we previously reported that PU.1 activates Phase 1–specific genes by recruiting RUNX1 to its binding sites (Hosokawa et al., 2018b; Ungerbäck et al., 2018). At the T-lineage commitment checkpoint, Bcl11b-mediated redirection of RUNX1 is essential for repressing alternative lineage-related genes and activating T lineage–specific genes, thereby reinforcing T cell fate (Hosokawa et al., 2018a). Therefore, the binding site selection of RUNX1 is regulated not only by the presence of the RUNX consensus motif but also by cell context–specific binding partners. Considering the importance of RUNX1-binding site selection in hematopoiesis, its expression levels must be tightly regulated to ensure competitive binding under limited RUNX1 protein availability across different developmental stages. Evidence for this hypothesis includes the observation that RUNX1 haploinsufficiency results in atypical hematopoiesis (Chin et al., 2016) and that a slight increase in RUNX1 protein levels can alter gene expression profiles during pro-T cell development (Shin et al., 2023). Hence, the dynamic redeployment of RUNX1 by developmental stage–specific interacting partners makes it one of the key regulating factors in hematopoiesis.

A portion of the data obtained in this paper have some technical limitations. First, the identification of RUNX1-interacting molecules was performed using an experimental system overexpressing tagged RUNX1. Therefore, we cannot rule out the possibility that we identified nonphysiological association with RUNX1. However, our ChIP-seq analysis identified genomic binding sites for endogenous RUNX1, CTCF, and Med12, and these results strongly support the organization of the LP-specific RUNX/CTCF complex. Secondly, deficiencies in RUNX, CTCF, or mediator complex components affected cell proliferation and survival. Indeed, preparing sufficient cell numbers for ChIP-seq analyses using *Cbfb*-, *Ctcf*-, or *Med12*-deficient cells was challenging. Consequently, the ChIP-seq analyses were performed using the minimum number of cells required, resulting in higher noise levels than usual and making detailed analysis difficult. In the future, genetic deletion systems that enable superior temporal control of targets, such as the degron system, may help to overcome this issue. Thirdly, current analysis techniques still do not fully capture the comprehensive, genome-wide correlation between TF-binding peaks and gene expression. In many cases, binding peaks for a specific TF are detected in the tens of thousands. Conversely, only a few hundred genes exhibit changes in expression when a specific TF is deleted. Multiple TF-binding sites can be found near a single gene; some are cobound by transcriptional activators, while others are co-occupied by transcriptional repressors. Furthermore, TFs often bind to enhancers or silencers located far from the gene and are involved in its transcriptional regulation. Current technology cannot accurately determine which binding peaks represent truly functional TF-binding sites or the genes they regulate. Although we succeeded in enriching functional complex–binding regions using stage-specific binding peaks of RUNX1, CTCF, Med12, and Notch1-IC, verifying whether all of these peaks are functional remains difficult. Incorporating higher order chromatin structure analysis and promoter–enhancer interaction studies in the future may enable more efficient identification of functional TF-binding peaks and their target genes.

In conclusion, we found that Notch stimulation induces the switching of RUNX1 protein complexes and genomic binding regions between the LP and Phase 1 stages. CRISPR/Cas9-mediated stage-specific deletion of RUNX factors and their binding partners revealed that the RUNX1/CTCF complex in LP negatively regulates T-signature gene expression, whereas the Notch1-IC/RUNX1/Mediator/p300 complex in Phase 1 serves as a positive regulator. This finding is consistent with the regulatory mechanism of hemocyte differentiation via the Notch/RUNX pathway observed in *Drosophila* (Terriente-Felix et al., 2013). Our results show that Notch-mediated functional conversion of RUNX factors through reorganization of protein complexes, and redeployment of genomic binding sites play a crucial role in the initiation of the T-lineage program (Fig. 8 G).

## Materials and methods

### Mouse experiments

*Ebf1*-deficient mice were provided by Dr. Rudolf Grosschedl (Max Planck Institute of Immunobiology and Epigenetics, Freiburg im Breisgau, Germany) (Lin and Grosschedl, 1995). B6.Cg-Tg(BCL2)25Wehi/J (Bcl2 transgenic [Tg], RRID:IMSR_JAX:002320) and B6.Gt(ROSA)26^Sortm1.1(CAG-cas9^_*_^,-EGFP)Fezh^/J (Rosa26-Cas9 knock-in, RRID:IMSR_JAX:026558) mice were purchased from the Jackson Laboratory. Both male and female 8- to 16-wk-old mice were used as cell sources for this study. Additionally, all the mice were bred and maintained under specific pathogen–free conditions at the animal facility of the Tokai University School of Medicine, at an ambient temperature of 21.5–24°C and 30–70% humidity, with lighting set as follows: 13 h on and 11 h off. The study protocol for animal experiments was reviewed and approved by the Institutional Animal Care and Use Committee at Tokai University; the approval numbers are as follows: 244007 and 241015.

### Cell culture of Cas9-LP cell lines

Cas9-LP cell lines were established from fetal liver progenitors of *Ebf1*-deficient Rosa26-Cas9 knock-in mice, as previously reported (Hirano et al., 2021). Cas9-LPs were cultured in Iscove’s Modified Dulbecco’s Medium (098-06465; Wako) supplemented with 10% fetal bovine serum (FBS; F7524; Sigma-Aldrich), penicillin–streptomycin–glutamine (Pen-Strep-Glutamine; 10378-016; Gibco), 50 μM β-mercaptoethanol (β-ME; 21985-023; Sigma-Aldrich), 10 ng/ml mouse stem cell factor (SCF, 250-03; PeproTech), 10 ng/ml human Fms-like tyrosine kinase 3 ligand (Flt3L; 300-19; PeproTech), and 10 ng/ml human IL-7 (200-07; PeproTech) in the presence of mitomycin C (139-18711; Wako)–treated OP9 cells (RRID:CVCL_4398). For T cell induction, Cas9-LPs were cocultured with OP9-DLL4 for 2–4 days under the same conditions used for Cas9-LP maintenance. OP9 and OP9-DLL4 cells were cultured in α-minimum essential medium (α-MEM; 137-17215; Wako) with 10% FBS, Pen-Strep-Glutamine, 50 μM β-ME. All cell lines were confirmed to be *Mycoplasma*-free before experiments.

### Cell culture of primary BM progenitors

BM cells were harvested from the femurs of 3- to 4-mo-old Rosa26-*Cas9* knock-in mice carrying the *Bcl2* transgene (Cas9;Bcl2 Tg). Suspensions of BM cells were stained for lineage (Lin) markers using biotin-conjugated lineage antibodies against CD11b (101204; BioLegend, RRID:AB_312787), CD11c (117304; BioLegend, RRID:AB_313773), Gr-1 (108404; BioLegend, RRID:AB_313369), TER-119 (116204; BioLegend, RRID:AB_313705), NK1.1 (108704; BioLegend, RRID:AB_313391), and CD3ε (100304; BioLegend, RRID:AB_312669). Cells were then incubated with anti-biotin magnetic beads (130-090-485; Miltenyi Biotec, RRID:AB_244365) and passed through a magnetic column using the “Deplete” program on an autoMACS separator (Miltenyi Biotec, RRID:SCR_018596) to isolate hematopoietic progenitors. The purified progenitors were infected with retroviral vectors encoding sgRNA and cultured in α-MEM, 20% FBS, 50 μM β-ME, Pen-Strep-Glutamine supplemented with 10 ng/ml of human IL-7, 10 ng/ml of mouse SCF, and 10 ng/ml of human Flt3L for 2 days. The cells were then transferred onto OP9-DLL1 stromal cells and cocultured for 3 days. After culture, cells were disaggregated, filtered through a 40-μm nylon mesh, and analyzed by FACSLyric (BD). Cells were stained with surface antibodies against CD45-PECy7 (103113; BioLegend, RRID:AB_312978), CD44-FITC (103005; BioLegend, RRID:AB_312956), CD25-APC-e780 (47-0251-82; eBioscience, RRID:AB_1272179), human NGFR-PE (345106; BioLegend, RRID:AB_2152647), and CD19-APC (17-0193-80; eBioscience, RRID:AB_1659678), and a biotin-conjugated lineage cocktail (CD8α; [13-0081-86; eBioscience, RRID:AB_466348], CD11b, CD11c, Gr-1, TER-119, NK1.1, TCRβ [109204; BioLegend, RRID:AB_313427], and TCRγδ [118103; BioLegend, RRID:AB_313827]) was used, followed by streptavidin-PerCPCy5.5 (405214; BioLegend, RRID:AB_2716577). Prior to cell surface staining, cells were treated with an Fc receptor blocker (130-059-901; Miltenyi Biotec, RRID:AB_2892112) to minimize nonspecific binding.

### scRNA-seq library preparation and sequencing

CD45^+^ Cas9-LPs, with or without Notch stimulation, were sorted using a FACSAria (BD) flow cytometer. scRNA-seq libraries were prepared using the 10x Genomics Chromium Next GEM Single Cell 3′ Reagent Kit and Library Construction Kit (10x Genomics), according to the manufacturer’s instructions. Briefly, cell suspensions were loaded onto the 10x Genomics Chromium Controller to generate gel beads in emulsions (GEMs). After GEM generation, samples were incubated at 53°C for 45 min in a thermal cycler (Veriti; Thermo Fisher Scientific) to generate polyA cDNA. Barcoding at the 5′ end was achieved by the addition of template switch oligonucleotides, which incorporated a unique cell barcode and unique molecular identifiers (UMIs). The GEMs were then broken, and single-stranded cDNA was purified using Dynabeads MyOne Silane Beads (Thermo Fisher Scientific). The purified cDNA was amplified (98°C for 3 min; [98°C for 15 s, 63°C for 20 s] × 11 cycles; 72°C for 1 min), and the cDNA quality was assessed using Agilent TapeStation. The cDNA was enzymatically fragmented, end-repaired, A-tailed, and subjected to double-sided size selection using SPRIselect beads (Beckman Coulter) Adapters provided with the kit were ligated to the cDNA fragments. A unique sample index for each library was introduced through 14 cycles of PCR amplification using the following conditions as mentioned in the kit (98°C for 45 s; [98°C for 20 s, 54°C for 30 s, and 72°C for 20 s] × 14 cycles; 72°C for 1 min). Indexed libraries were subjected to a second round of double-sided size selection and quantified, and their quality was assessed using the Agilent TapeStation. Libraries were submitted to GeneWiz for clustering on a NovaSeq paired-end read flow cell, and sequenced on Read 1 (R1: 10× barcode and UMIs), followed by eight cycles of the I7 index (sample index) and 89 cycles based on R2 (transcript). Raw sequencing data were processed using the 10× Genomics Cell Ranger Single Cell Software Suite (10x Genomics). This pipeline performed sample demultiplexing, alignment to the mouse reference genome (*mm10*), filtering, UMI counting, and single-cell 3′-end gene expression quantification according to the manufacturer’s parameters.

### scRNA-seq data analysis

The R package Seurat (v4.1.2) was used to analyze the scRNA-seq data, performing clustering of cells in a merged matrix. Cells were filtered based on the following criteria to remove low-quality cells and potential doublets: cells expressing fewer than 200 genes or more than 1,600 genes were considered low-quality and removed. Cells expressing more than 6,000 genes were filtered out as potential doublets. Cells with a mitochondrial gene expression >10% were excluded. The gene expression counts for each cell were normalized by dividing each gene count by the total gene counts per cell, multiplying by a scale factor of 10,000, and applying a natural log transformation. The FindVariableFeatures function was used to select variable genes using default parameters. The ScaleData function was used to scale and center the counts in the dataset. Principal component analysis was performed on the variable genes using 50 principal components for downstream clustering and UMAP dimensionality reduction at a resolution of 0.5. Cluster markers were identified using the FindAllMarkers function, and cell types were manually annotated based on known marker genes. DEGs between groups were identified using Welch’s *t* test (P < 0.05). The cluster markers defined by FindAllMarkers were used as DEGs in each cluster. Cell cycle phases (G1, S, and G2/M) were identified using the CellCycleScoring function, based on cell cycle–related gene expression. Pseudo-time trajectory analysis was performed using Monocle3 to assess maturation stages. Coregulated genes were grouped into modules using Louvain community analysis in Monocle3 allowing the assessment of correlation between clusters and gene sets. TF activity was calculated using SCENIC based on gene regulatory network analysis.

### Two-step affinity purification of RUNX1 complexes from LPs and mass spectrometry

Cas9-LPs were infected with either a mock control (pMxs-IRES-hNGFR) or Myc-FLAG-RUNX1-ERT2–containing retroviruses. After 4-h co-incubation with the retrovirus, Myc-FLAG-tagged RUNX1-ERT2–infected hNGFR^+^ cells were transferred onto OP9 or OP9-DLL4 cells. 3 days after infection, the cells were treated with 200 nM 4-hydroxytamoxifen (T2859; Sigma-Aldrich) for 6 h, and then, 3 × 10^8^ cells were solubilized using an immunoprecipitation buffer containing protease inhibitors (50 mM Tris-HCl (pH 7.5), 150 mM NaCl, 10% glycerol, 0.1% Tween, 1 mM EDTA, 10 mM NaF, 1 mM dithiothreitol, and a protease inhibitor cocktail [Roche Applied Science]) and lysed on ice for 30 min with gentle shaking and then sonicated using a VP-55 sonicator (TAITEC) for three cycles at amplitude 6 for 20 s, followed by 1-min rest between cycles. After lysis, insoluble materials were removed by centrifugation, and the supernatant was subjected to immunoprecipitation with anti-FLAG M2 agarose (A2220; Sigma-Aldrich, RRID:AB_10063035) overnight at 4°C. The immune complexes were eluted from agarose beads using 3×FLAG peptide (Sigma-Aldrich), and then subjected to a second immunoprecipitation step using anti-Myc magnetic beads (MBL). The immune complexes were eluted from the magnetic beads with MBL and separated by SDS-PAGE. The protein bands were excised from the gel and subjected to mass spectrometric analysis to identify the corresponding proteins. Gel pieces were washed twice with 100 mM bicarbonate in acetonitrile, and the proteins were digested with trypsin. The resulting peptides were treated with 0.1% formic acid and analyzed by LC-MS/MS using an Advance ultra-high-performance liquid chromatograph (Bruker) coupled with an Orbitrap Velos Pro mass spectrometer (Thermo Fisher Scientific). The resulting tandem mass spectrometry dataset was analyzed using the Mascot software program (Matrix Science), where a Mascot score >100 indicated a probability of <1e−10 that the observed match was a random event.

### Cloning

For CRISPR targeting, the sgRNA expression vector (E42-dTet) was used as previously described (Hosokawa et al., 2018b). 20-mer sgRNAs were designed using the Benchling web tool (https://www.benchling.com) and inserted into the empty sgRNA expression vector by PCR-based insertion. Three sgRNA expression vectors were generated for each gene, and pooled retroviral plasmids were used to generate retroviral supernatant. The sgRNA sequences used in this study are listed below:

sgControl (Luciferase) #1: 5′-ACC​GCG​AAA​AAG​TTG​CGC​GG-3′
sgControl (Luciferase) #2: 5′-GGC​ATG​CGA​GAA​TCT​CAC​GC-3′
sgCbfb #1: 5′-ACA​GCG​ACA​AAC​ACC​TAG​CC-3′
sgCbfb #2: 5′-CGT​GTC​TGG​CGC​TCC​TCG​TG-3′
sgCbfb #3: 5′-GAG​GAG​CAA​GTT​CGA​GAA​CG-3′
sgCTCF #1: 5′-GAT​ATG​GCC​TTT​GTG​ACC​AG-3′
sgCTCF #2: 5′-CCA​CAC​AAA​TGC​CAT​CTG​TG-3′
sgCTCF #3: 5′-GAT​GAA​GAC​TGA​AGT​CAT​GG-3′
sgMed12 #1: 5′-ATG​GCC​ATC​TCT​ACA​TCA​TG-3′
sgMed12 #2: 5′-TCT​TGA​GGG​TAC​ACA​TCG​GG-3′
sgMed12 #3: 5′-ACG​GCT​TTG​AAT​GTA​AAA​CA-3′

### CRISPR/Cas9-mediated deletion of target genes in LPs

The Cas9-LPs were transduced with retroviral vectors encoding sgRNA-hNGFR and transferred onto OP9 or OP9-DLL4 cells for 3–5 days after infection. Following this, the CD25 and CD44 profiles of the sgRNA^+^ retrovirus-infected cells were analyzed. For QuantSeq 3′ mRNA sequencing, Cas9-LPs were infected with sgRNA-hNGFR. 5 days after sgRNA transduction, sgRNA-introduced hNGFR^+^CD45^+^ LPs were sorted using a BD FACSAria flow cytometer.

### Flow cytometry

For staining of sgRNA-introduced Cas9-LPs, surface antibodies against CD44-FITC (103005; BioLegend, RRID:AB_312956), CD25-eFluor 450 (48-0251-82; eBioscience, RRID:AB_10671550), CD45-PECy7 (103113; BioLegend, RRID:AB_312978), and human NGFR-PE (345106; BioLegend, RRID:AB_2152647) were used. All cells were analyzed using a FACSLyric (BD), FACSAria Fusion (BD), or LSRFortessa (BD) flow cytometer with FlowJo software (Tree Star).

### Immunoblotting

Cytoplasmic and nuclear extracts were prepared using NE-PER Nuclear and Cytoplasmic Extraction Reagents (Pierce). Lysates were run on 7.5% or 10% polyacrylamide gels, followed by immunoblotting. The antibodies used for immunoblot analysis were anti-tubulin-α (T6199; Sigma-Aldrich, RRID:AB_477583), anti-lamin B (13435; CST, RRID:AB_2737428), anti-Myc (M192-3; MBL, RRID:AB_11160947), anti-FLAG (F3165; Sigma-Aldrich, RRID:AB_259529), anti-Cbfb (62184; CST, RRID:AB_2722525), anti-CTCF (3418; CST, RRID:AB_2086791), anti-Med12 (A300-774A; Bethyl, Boston, RRID:AB_669756), and anti-Notch1-IC (4147; CST, RRID:AB_2153348).

### ChIP-seq

For ChIP-seq analysis, Cas9-LPs (3–10 × 10^6^) with or without Notch stimulation were used. Cells were fixed with 1 mg/ml disuccinimidyl glutarate (Thermo Fisher Scientific) in phosphate-buffered saline (PBS) for 30 min at 25°C followed by an additional 10-min fixation with formaldehyde (final concentration: 1%). The reaction was quenched by adding 1/10 the volume of 0.125 M glycine, and the cells were washed with cold PBS. Pelleted nuclei were dissolved in lysis buffer (0.5% SDS, 10 mM EDTA, 0.5 mM EGTA, 50 mM Tris-HCl [pH 8.0], and protease inhibitor cocktail). Chromatin was sheared using a Bioruptor (Diagenode, RRID:SCR_023470) for 18 cycles (30-s sonication followed by 30-s rest, high power). Five micrograms of antibodies against RUNX1 (Abcam, ab23980, RRID:AB_2184205), CTCF (3418; CST, RRID:AB_2086791), Med12 (A300-774A; Bethyl, RRID:AB_669756), or p300 (57625; CST, RRID:AB_3068009) were prebound to Dynabeads coated with anti-rabbit IgG and incubated overnight at 4°C with the chromatin complexes. The samples were then washed and eluted overnight at 65°C in ChIP elution buffer (20 mM Tris-HCl, pH 7.5, 5 mM EDTA, 50 mM NaCl, 1% SDS, and 50 μg/ml proteinase K). Purified chromatin fragments were cleaned using ChIP DNA Clean & Concentrator (D5205; Zymo).

ChIP-seq libraries were constructed using NEBNext Ultra II DNA Library Prep Kit (E7645S; NEB) with Sample Purification Beads, and NEBNext Multiplex Oligos for Illumina (E6440S; NEB). Libraries were sequenced on an Illumina NextSeq 2000 in paired-end read mode with a read length of 50 nt. Base calling was performed using Real-Time Analysis (RTA) 4.12.2, followed by conversion of the reads to FASTQ format using bcl2fastq v2.20.0.422, generating ∼30 million reads per sample. ChIP-seq data were mapped to the mouse genome (NCBI37/mm10) using Bowtie (v1.1.1; https://www.encodeproject.org/software/idr/) with “-v 3 -k 11 -m 10 -t -best -strata” settings, and HOMER tag directories were created with makeTagDirectory and visualized in the UCSC Genome Browser (https://genome.ucsc.edu). ChIP peaks were identified with findPeaks.pl against a matched control sample using the setting “-P.1 -LP.1 -poisson.1 -style factor.” The identified peaks were annotated to genes using the annotatePeaks.pl with the mm10 genomic build in the HOMER package. Peak reproducibility was determined using the HOMER adaptation of the Irreproducibility Discovery Rate (IDR) package, according to the ENCODE guidelines (https://sites.google.com/site/anshulkundaje/projects/idr). Only the reproducible high-quality peaks with a normalized peak score ≥15 were considered for further analysis. Motif enrichment analysis was performed using the findMotifsGenome.pl command in HOMER with a 200-bp window. Tag density plots and heatmaps were created with *annotatePeaks.pl* (*–hist* or *–hist* & *-ghist*, respectively) in a 2,000-bp region surrounding the indicated TF peak center, and by hierarchical clustering of the tag count profiles in Cluster3 with average linkage followed by TreeView visualization. GO analysis was performed using the Genomic Regions Enrichment of Annotations Tool (GREAT) analysis tool (https://great.stanford.edu/public/html/).

### QuantSeq 3′ mRNA sequencing

Total RNA was isolated from 3 × 10^5^ cultured cells using the RNeasy Micro Kit (74004; Qiagen). 3′mRNA library was prepared from the total RNA (500 ng) with QuantSeq 3′ mRNA-seq Library Prep Kit FWD (LEXOGEN) according to the manufacturer’s instructions. After PCR amplification, the size distribution and yield of the library were determined using the D1000 High Sensitivity TapeStation (Agilent Technologies). The pooled libraries were loaded onto the Illumina NextSeq 2000 platform and analyzed by 75-bp single-end reads. Adapter sequences were trimmed from the raw RNA-seq reads with fastp. The trimmed reads of each sample were mapped to the reference mouse genome (*mm10*) using Spliced Transcripts Alignment to a Reference and normalized to one million reads in the original library. DEGs were defined as adjusted P < 0.05, |log_2_FC| > 1, and TPM > 10 based on measurements from three biologically independent replicates for each sample type.

### Statistical analyses

To compare the average of two groups, a two-sided *t* test was performed (Fig. 7 A). Additionally, a one-way analysis of variance (ANOVA) with Dunnett’s multiple comparisons was performed to compare the average of treatment groups and control groups (Fig. 4, C, D, F, and G; Fig. 8, B, C, E, and F; and Fig. S5, C and E). A chi-square test with Yates’s correction was conducted to evaluate the association between categorical variables (Fig. S5, A and B). Parametric statistical tests were performed when the data met the assumptions of normality. The normality of the data was tested by the Shapiro–Wilk test. Equal variances were tested by Levene’s test formally, and data that did not meet equal variances were handled with adjustment, such as Welch’s correction. One-way ANOVA, two-sided *t* test, and chi-square test with Yates’s correction were performed using Prism software (v.9.5.1, GraphPad): *P < 0.05; **P < 0.01 for *t* test and one-way ANOVA.

### Online supplemental material

Fig. S1 shows T cell development status of Cas9-LPs after Notch stimulation *in vitro* and *in vivo* and key TF expressions in bulk transcriptome and proteome data. Fig. S2 shows key TF expressions and cell cycle status in scRNA-seq data, mRNA levels of key TFs in LP and DN subsets, heatmaps for RUNX1 ChIP signals, heatmaps for motif analysis of RUNX1-binding sites, nuclear translocation of Myc-FLAG-RUNX1-ERT2 by tamoxifen treatment, and summary of Mascot scores for RUNX1-interacting molecules. Fig. S3 shows validation of *Cbfb*, *Ctcf*, and *Med12* disruption by immunoblotting, volcano plots of transcriptome data, and bidirectionally regulated genes by RUNX factors. Fig. S4 shows heatmaps for RUNX1, CTCF, representative LP-specific RUNX1 peaks around the T-signature loci and their expression, Med12, characterization of Med12 ChIP peaks, and Notch-IC–binding changes before and after Notch stimulation, and Venn diagrams for Notch-IC and RUNX1-binding genomic regions. Fig. S5 shows statistical analysis of LP-specific RUNX1/CTCF-binding sites and Phase 1–specific RUNX1/Notch1-IC–binding peaks, percentages of CD19^+^ and Lin^−^ cells in *Cbfb*-, *Ctcf*-, or *Med12*-deficient cells, and progression of B cell differentiation. Table S1 shows the list of RUNX1-interacting molecules. Table S2 shows the list of RUNX-regulated DEGs.

## Supplementary Material

Table S1 shows the list of RUNX1-interacting molecules.

Table S2 shows the list of RUNX-regulated DEGs.

SourceData F2 is the source file for Fig. 2.

SourceData FS2 is the source file for Fig. S2.

SourceData FS3 is the source file for Fig. S3.

## Data Availability

All the new deep-sequencing data reported in this paper are publicly available through the NCBI GEO accession numbers GSE291464, GSE291465, and GSE296265. Other data will be provided by the lead corresponding author upon reasonable request.
