# Supplementary material for: Notch interaction with RUNX factors regulates initiation of the T-lineage program
Source: J Exp Med. 2025 Dec 4;223(2):e20250911. doi: 10.1084/jem.20250911 (PMC12677141; doi:10.1084/jem.20250911)
Supplement: SourceData F2 — is the source file for Fig. 2. [file jem_20250911_sourcedataf2.pdf]

Fig. 2E

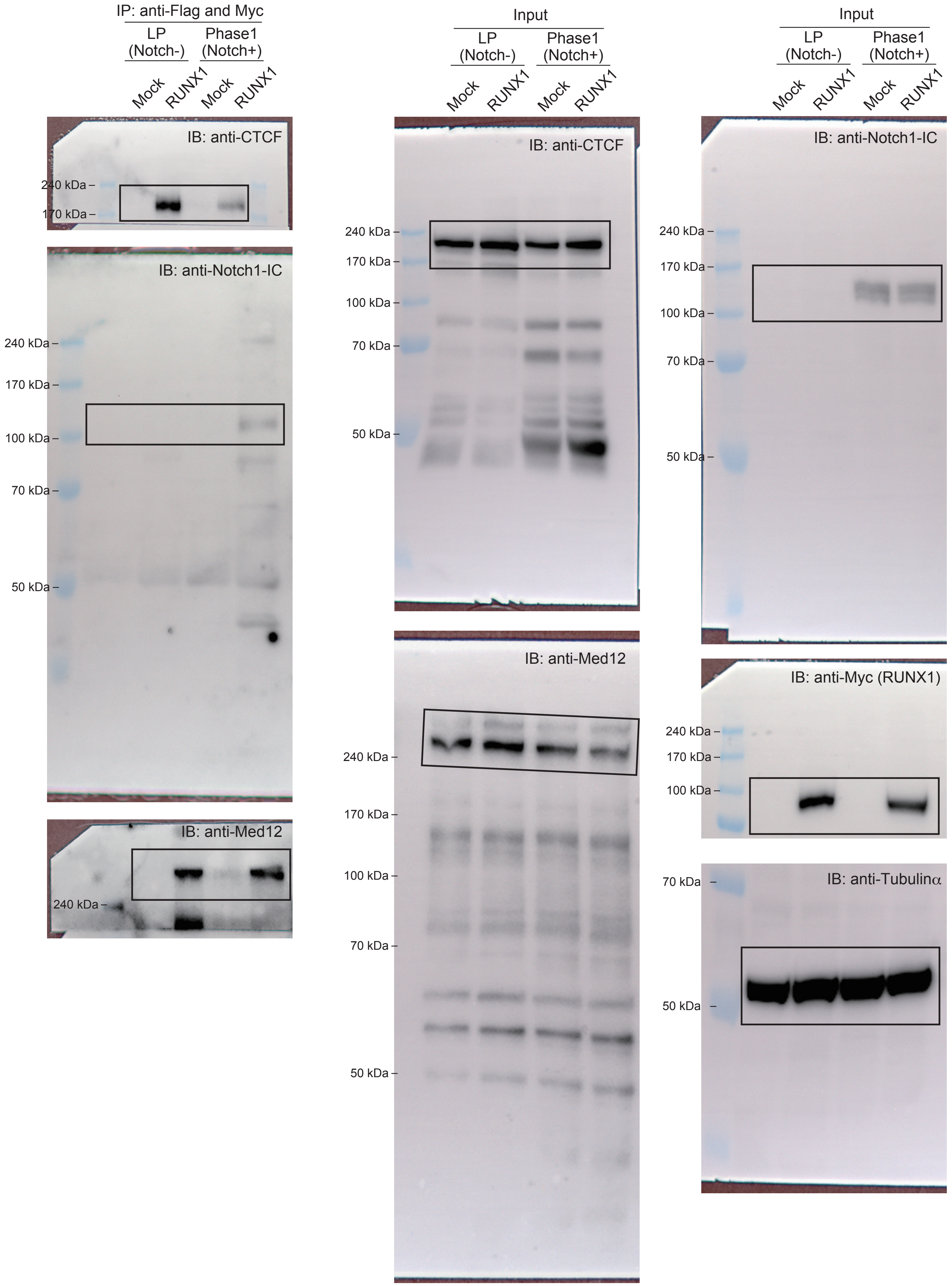

IP: anti-Flag and Myc
LP (Notch-)
Phase1 (Notch+)
Mock
RUNX1
Mock
RUNX1
IB: anti-CTCF
240 kDa
170 kDa
IB: anti-Notch1-IC
240 kDa
170 kDa
100 kDa
70 kDa
50 kDa
IB: anti-Med12
240 kDa
Input
LP (Notch-)
Phase1 (Notch+)
Mock
RUNX1
Mock
RUNX1
IB: anti-CTCF
240 kDa
170 kDa
100 kDa
70 kDa
50 kDa
IB: anti-Med12
240 kDa
170 kDa
100 kDa
70 kDa
50 kDa
Input
LP (Notch-)
Phase1 (Notch+)
Mock
RUNX1
Mock
RUNX1
IB: anti-Notch1-IC
240 kDa
170 kDa
100 kDa
70 kDa
50 kDa
IB: anti-Myc (RUNX1)
240 kDa
170 kDa
100 kDa
IB: anti-Tubulinα
70 kDa
50 kDa
