# Supplementary figures and images for: Notch interaction with RUNX factors regulates initiation of the T-lineage program
Source: J Exp Med. 2025 Dec 4;223(2):e20250911. doi: 10.1084/jem.20250911 (PMC12677141; doi:10.1084/jem.20250911)

Fig. S2H

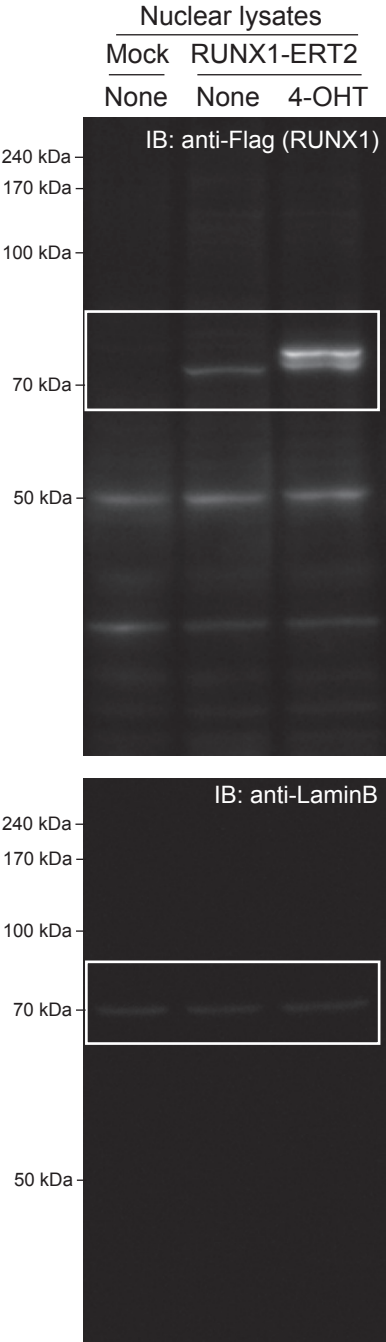

Supplement: SourceData FS2 — is the source file for Fig. S2. [file jem_20250911_sourcedatafs2.pdf]

Fig. S3A

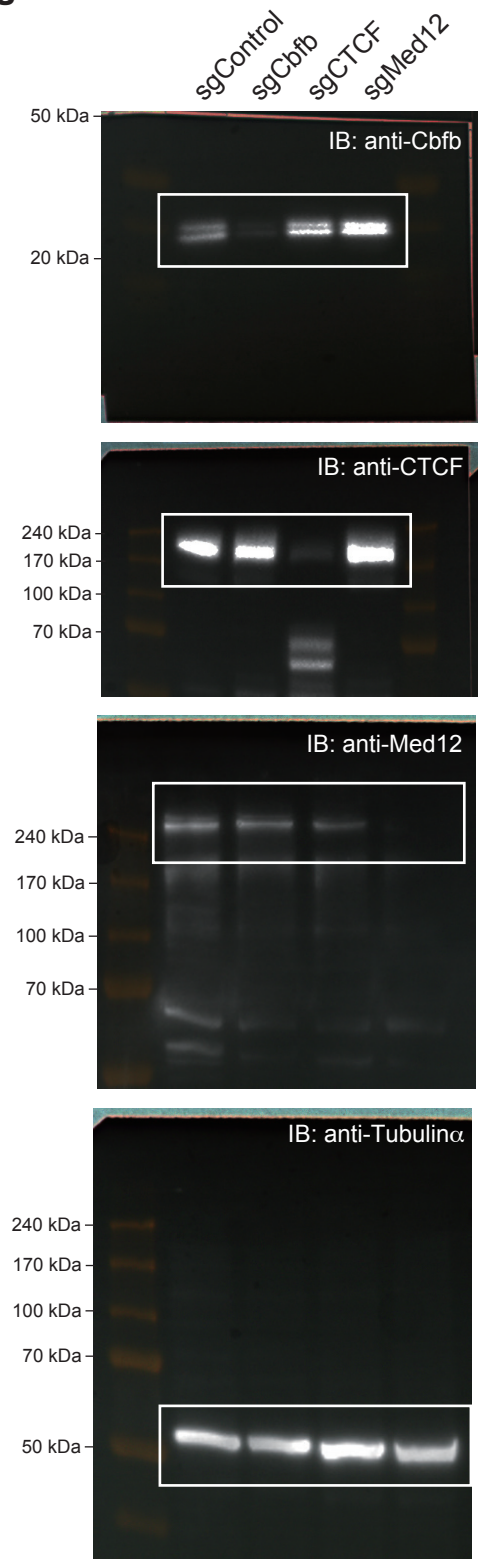

Supplement: SourceData FS3 — is the source file for Fig. S3. [file jem_20250911_sourcedatafs3.pdf]
